# Bridging the gap: exploring the causal relationship between metformin and tumors

**DOI:** 10.3389/fgene.2024.1397390

**Published:** 2024-06-19

**Authors:** Zexin Zhang, Wenfeng Wu, Zexia Wu, Yihan He, Xuesong Chang, Shenyuan Deng, Rui Zhou, Yadong Chen, Haibo Zhang

**Affiliations:** ^1^ The Second Clinical School of Guangzhou University of Chinese Medicine, Guangzhou, China; ^2^ The Second Affiliated Hospital of Guangzhou University of Chinese Medicine, Guangzhou, China; ^3^ Guangdong Provincial Key Laboratory of Clinical Research on Traditional Chinese Medicine Syndrome, Guangzhou, China; ^4^ Guangdong-Hong Kong-Macau Joint Lab on Chinese Medicine and Immune Disease Research, Guangzhou University of Chinese Medicine, Guangzhou, China; ^5^ State Key Laboratory of Dampness Syndrome of Chinese Medicine, The Second Affiliated Hospital of Guangzhou University of Chinese Medicine, Guangzhou, China

**Keywords:** causal relationship, metformin, tumors, phenome-wide Mendelian randomization, meta-analysis

## Abstract

**Objective:**

Numerous studies have reported that metformin can reduce the risk of tumor development. However, some of the results of these studies are conflicting, necessitating a more reliable evaluation.

**Methods:**

We conducted a Mendelian randomization phenome-wide association study (MR-PheWAS) of tumors to explore the causal relationship between metformin and tumors. Two cohorts of patients taking metformin were obtained from the UK Biobank. Complete phenotype data of the tumors were obtained from FinnGen_R10. We elucidated the causal relationship using a two-sample Mendelian randomization (MR) analysis. More importantly, we conducted a meta-analysis to ensure relatively unbiased results. In the MR analysis, we used the inverse-variance weighted (IVW) method as the main outcome indicator. Subsequently, two cohorts were integrated for the meta-analysis. Finally, we investigated the mechanisms through mediational MR analysis.

**Results:**

MR analysis revealed that metformin might have a causal relationship with 13 tumor-associated phenotypes in the training cohort. Four phenotypes were validated in the testing cohort. In the training and testing cohorts, metformin exhibited a protective effect against brain meningiomas and malignant neoplasms of the breast (HER-positive), oral cavity, tonsils, and the base of the tongue. Intriguingly, after integrating the results of the two cohorts for the meta-analysis, 12 results were statistically significant. Mediational MR analysis suggested that the effects of metformin on brain meningiomas may be weakened by the presence of the family Oxalobacteraceae.

**Conclusion:**

Metformin exhibits potential preventive and therapeutic effects on four types of tumors: brain meningioma, malignant neoplasms of the breast (HER-positive), oral cavity and tonsils, and the base of the tongue. Large randomized controlled trials are required to confirm these findings.

## Background

Malignant tumors represent the most significant threat to human health and survival worldwide (Wang et al., 2020). According to the World Health Organization (WHO) 2022 report, malignant tumors account for approximately 10 million deaths globally, constituting one-sixth of the total deaths (World Health Organization, 2022a). Approximately 19.29 million new cancer cases were reported in 2020, of which 10.06 million were among men and 9.23 million were among women. The high mortality rate and growing number of patients with cancer have imposed a significant burden on the global economy. Data have demonstrated that approximately $1.2 trillion is needed to treat tumor patients annually, nearly 2% of the world’s gross product in 2019 (Wild and Stewart, 2020). Therefore, it is imperative to improve the quality of life and prolong the survival time of patients with cancer while reducing the medical and economic burden.

As research on immune checkpoint inhibitors (ICIs) and antibody-drug conjugates (ADCs) continues to advance, many patients will benefit from new therapies. Data indicate that the survival time of these patients can be significantly prolonged (Robert, 2020; Fu et al., 2022). For example, the 5-year overall survival (OS) rate of patients with non-small cell lung cancer (NSCLC) who received programmed cell death protein-1 (PD-1) inhibitors as second-line treatment rather than chemotherapy increased from 13% to 25% (Marei et al., 2023). In contrast, it peaked at 32% for first-line treatment (Xu et al., 2019). For patients with unresectable or metastatic HER2-positive breast cancer, DESTINY-Breast01 demonstrated that DS-8201a could achieve an overall response rate (ORR) of 60.3% (95% CI: 52.9, 67.4), with a median duration of response (DoR) of 14.8 months (Narayan et al., 2021). Although ICIs and ADCs have brought significant benefits to cancer patients, it is important to note that these drugs form an impregnable barrier that prevents their effective administration to cancer patients. Developing anti-tumor drugs is often costly, with one study indicating a median cost of $648 million (Prasad and Mailankody, 2017). Adverse effects present an additional challenge for patients. Therefore, the balance between efficacy and price is a key issue that needs to be addressed for future use of oncology medicines.

Diabetes mellitus is associated with an increased risk of cancer incidence and mortality (Tseng, 2004). Metformin is a treatment of choice for patients with type two diabetes mellitus (T2DM) (Feig, 2012). The use of this drug has a history spanning over 60 years since its inception in the 1950s (Tang et al., 2018). Metformin is widely used to treat T2DM because of its significant efficacy and low price (American Diabetes Association, 2015). The mechanism of action of metformin is mainly related to its inhibition of intestinal glucose absorption and increase in glucose uptake and utilization in the peripheral tissues (Bowden et al., 2015).

Interestingly, many recent studies have explored the therapeutic effects of metformin in tumors, implying that metformin may have potential anti-tumor effects. Research has indicated that the anti-tumor effects of metformin are mainly due to the activation of AMP-activated protein kinase (AMPK) and inhibition of mammalian targets of rapamycin (mTOR) (Dowling et al., 2007). Furthermore, the intestinal flora is closely related to malignant intestinal tumors, and metformin regulates the intestinal flora and improves the intestinal barrier (Ursini et al., 2018), which is also one of the potential mechanisms by which metformin exerts its anti-tumor effects. However, not all results support this notion. MA.32 was a phase III randomized trial that enrolled over 3,600 patients with high-risk operable breast cancer and randomized them to receive either 850 mg of metformin or a placebo for 5 years. Unfortunately, the addition of adjuvant metformin did not improve the disease-free survival (DFS) of patients with breast cancer, estrogen receptor-positive or estrogen receptor-negative (Goodwin et al., 2022).

Similarly, two clinical trials evaluated the combined use of metformin in patients with NSCLC, indicating no benefit (Skinner et al., 2021; Tsakiridis et al., 2021). However, an analysis of Asian populations revealed that metformin use in newly diagnosed T2DM patients could significantly reduce the incidence of lung cancer (Tseng, 2017). Although some studies have confirmed the potential anti-tumor effects of metformin, its underlying mechanisms often manifest in diverse ways. Retrospective or prospective studies are susceptible to confounding variables, which may challenge a reliable result. Furthermore, some studies have reported conflicting results. Therefore, using relatively reliable methods to evaluate the causal relationship between metformin and tumors complementarily is imperative and requires resolution.

Mendelian randomization (MR) is an emerging epidemiological research method that uses genetic variants strongly correlated with exposure factors as instrumental variables (IVs) to evaluate the causal relationship between exposure factors and outcomes (Richmond and Davey Smith, 2022). Because the screening of IVs is rigorous, they can represent the research variables to infer causal relationships. One notable advantage is its ability to significantly minimize the influence of confounding variables and reverse causality in observational studies, thus increasing the reliability of the findings (Davies et al., 2018).

In summary, this study used tumor-associated MR-PheWAS to reveal the causal relationship between metformin and tumors and to validate the results using a testing cohort. After integrating the results of the two cohorts, we performed a meta-analysis to improve the evidence level of the outcome. Finally, a mediating MR analysis was conducted to elucidate the potential mechanisms of metformin-mediated tumor risk.

## Materials and methods

### Data sources and acquirement

The GWAS summary data for metformin came from the UK biobanks and were obtained from the ieu opend gwas project (https://gwas.mrcieu.ac.uk/). The GWAS summary datasets were organized into 18 batches. These two cohorts are from the Neale lab analysis of UK Biobank phenotypes, round 1 (ukb-a) (Elsworth et al., 2023), and IEU analysis of UK Biobank phenotypes (ukb-b). We used one of the cohorts as the training cohort and the other as the validation cohort from two different consortiums: MRC-IEU and Neale Lab.

Tumor-associated phenome-wide data were acquired from the Finnish biobanks (Kurki et al., 2023). The FinnGen study is a large-scale genomics initiative correlating genetic variation with health data to understand disease mechanisms and predispositions by analyzing over 500,000 Finnish biobank samples. The project is a joint venture between Finnish research organizations, biobanks, and international industry partners. We extracted relevant tumor phenotypes from the FinnGen R10 data using “cancer” as the keyword. FinnGen R10 contains the genetic data for 412,181 samples and 2,408 phenotypes.

### Screening of instrumental variables (IVs)

The criteria for screening IVs followed the three assumptions of MR analysis. The three assumptions of MR are correlation, exclusivity, and independence. To ensure that the selected IVs were highly correlated with exposure, the *p*-value was defined as < 5e^−08^. Among outcomes, *p* values for IVs were defined as < 5e^−06^ (Korologou-Linden et al., 2022). In addition, linkage disequilibrium is not allowed in MR analysis, which will challenge the reliability of the results. Therefore, we also screened for IVs by setting *r* ^2^ = 0.001 and kb = 10,000 (Zhang et al., 2023). We also used the Steiger test to remove the IVs with reverse causality. Finally, an F-test was conducted to determine the strength of the IVs. This study defined IVs with F > 10 as strong instrumental variables. The formula of the F-test was as follows: F = (Beta/Se) ^ 2 (Bowden et al., 2019).

In general, the exclusivity and independence assumptions require that IVs only affect outcomes through exposure and cannot be confounded with other factors that affect outcomes. IVs with horizontal pleiotropy are prohibited in MR analysis as they typically indicate that the IVs have more than one genetic function, violating the assumptions of MR analysis. Therefore, we used MR-Egger and Presso methods to test the screened IVs (Bowden et al., 2015; Verbanck et al., 2018). Only the IVs that met both tests were proven to have no horizontal pleiotropy. Otherwise, the IVs with horizontal pleiotropy were eliminated.

### Tumor-associated phenome-wide Mendelian randomization analysis in the training cohort

The two-sample MR (TSMR) method analyzed the causal relationship between metformin and tumor-associated phenome-wide. In the TSMR analysis, we used four methods simultaneously: inverse-variance weighted(IVW), MR-Egger, weighted median model, and weighted model. Among these, the IVW method is the primary method used for causal relationship analysis. A *p*-value of < 0.05 for the IVW method was considered to be suggested. IVW is crucial for evaluating causal relationships in MR analysis (Burgess et al., 2015). A distinguishing feature of this method is the exclusion of the intercept term in the regression analysis and the use of the inverse of the outcome variance as a weight for fitting purposes. Like the IVW method, MR-Egger uses the reciprocal of the outcome variance as a weighting factor for regression fitting (Bowden et al., 2015). However, a notable distinction lies in including the intercept term during regression, enabling the concurrent evaluation of pleiotropy within MR-Egger’s framework.

Furthermore, multiple testing corrections are essential steps in statistical hypothesis testing. We usually consider *p* < 0.05 as a threshold to determine the significance. However, simultaneous comparisons of multiple data sets in a study may introduce random effects that cause some data to exceed the threshold, resulting in false-positive results. The greater the number of tests, the greater the probability of false positives. Therefore, we used the False Discovery Rate(FDR) to perform multiple corrections on the *p*-value of IVW (Benjamini and Hochberg, 1995). The *p*-value of FDR < 0.05 was considered to be statistically significant.

### Sensitivity analysis and heterogeneity testing

The stability of IVs is an important factor in MR analysis, which usually requires that all IVs equally contribute to the outcome, ensuring that the results are not dominated by a single IV. Therefore, a leave-one-out sensitivity analysis was performed on all IVs in the MR analysis. The leave-one-out sensitivity analysis evaluates the effect size of the remaining IVs on the outcome by eliminating certain IVs individually. Additionally, we tested for heterogeneity using Cochran’s Q statistic for all IVs (Burgess et al., 2013). A random effects model was used for IVs with significant heterogeneity; otherwise, a fixed effects model was used.

### Verification based on testing cohort

Another cohort was selected to verify the reliability of the MR analysis results. The testing cohort was analyzed as described for the training cohort.

### Meta-analysis based on IVW method of training cohort and testing cohort

To further consolidate the reliability of the MR analysis results, we conducted a meta-analysis to integrate the findings of the IVW method based on training and testing cohorts. I^2^ was used to detect heterogeneity in the meta-analysis. A fixed effects model was used for I^2^ ≤ 50% and *p* ≥ 0.05, whereas a random effect model was used for I^2^ > 50% and *p* < 0.05 (Higgins et al., 2003).

### Mediational MR analysis

To further explore the potential mechanisms by which metformin affects tumor risk, we performed mediated MR analysis. First, we performed pairwise TSMR analysis between metformin, intestinal flora, lipid metabolism, immune cells, and positive tumor phenotypes. The data on intestinal flora come from Kurilshikov A, published in Nature Genetics in 2021 (Kurilshikov et al., 2021). Our previously published article describes the data and screening thresholds (Zhang et al., 2023). The data on lipid metabolism come from Ottensmann L, published in Nature Communications in 2023 (Ottensmann et al., 2023). Data on immune cells come from Orrù V, published in Nature Genetics in 2020 (Orrù et al., 2020). Phenotypes with significant differences in pairwise TSMR analyses were retained for further studies.

A test for mediation MR analysis was conducted using the coefficient product method. In the mediation MR analysis, the effect size of metformin on gut microbiota, lipid metabolism, and immune cells was recorded as a, the effect size of gut microbiota, lipid metabolism, and immune cells on the positive tumor phenotype was recorded as b, and the effect size of metformin on the positive tumor phenotype was recorded as c’. The effect size of the mediator was obtained by calculating a × b. A direct effect was obtained using c’—a × b. The proportion of the mediating effect to the total effect was obtained using a × b/c’. The Sobel test was used to determine whether there was a significant difference in the mediation effect (http://quantpsy.org/sobel/sobel.htm).

## Results

### Data sources and acquirement

Two metformin queues were obtained from the GWAS summary data, and their IDs were ukb-a-159 and ukb-b-14609. Among these, ukb-a-159 was used as the training cohort, and ukb-b-14609 was used as the testing cohort. ukb-a-159 contained 10,894,596 SNPs and 337,159 samples, including 8,392 cases and 328,767 controls. ukb-b-14609 comprised 9,851,867 SNPs and 462,933 samples, including 11,552 cases and 451,381 controls. The two cohorts were from Europe and included both males and females.

A total of 129 tumor-associated phenotypes were obtained from 2,408 phenotype data from the FinnGen R10 dataset. These phenotypes summarize the different types of tumors, including malignant neoplasms of the adrenal gland, acute lymphocytic leukemia, malignant neoplasm of the anus, brain astrocytoma, and basal cell carcinoma.

### Screening of IVs

A total of 26 SNPs were extracted from ukb-a-159 and used as exposed IVs. Finally, 13 phenotypic SNPs were extracted from the 129 tumor-associated phenotypes. Among them, brain astrocytoma contained 24 SNPs, HER-positive malignant neoplasm of the breast contained 23 SNPs, malignant neoplasm of the breast contained 22 SNPs, malignant neoplasm of bronchus and lung contained 23 SNPs, colon adenocarcinoma contained 24 SNPs, malignant neoplasm of colon contained 24 SNPs, colorectal adenocarcinoma contained 24 SNPs, colorectal cancer contained 24 SNPs, non-small cell lung cancer contained 24 SNPs, brain meningioma contained 24 SNPs, malignant neoplasm of oral cavity contained 24 SNPs, a cancer of tonsil and base of tongue contained 24 SNPs and carcinoma *in situ* of the vulva contained 24 SNPs. In this study, the F values of all the IVs were >10, and there were no weak IVs. The details of the IVs are presented in Supplementary Table S1. The Steiger test did not identify any IVs with reverse causality. The MR-Egger and Presso test removed rs34872471 in the malignant neoplasm of the breast and rs4932264 in the malignant neoplasm of the bronchus and lung with horizontal pleiotropy.

### Tumor-associated phenome-wide MR analysis in the training cohort

**FIGURE 1:**
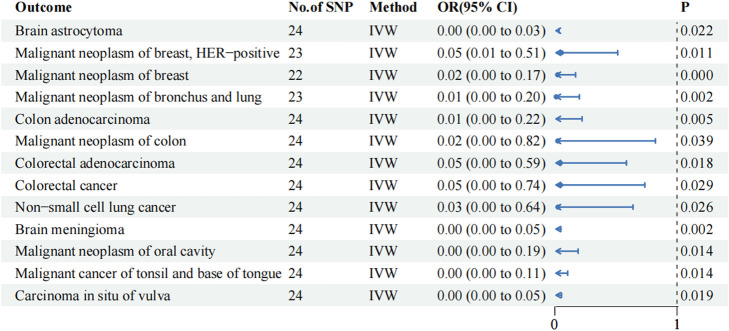
IVW results of metformin against 13 tumor-associated phenotypes in the training cohort. The blue dots represent the OR value, the blue solid line represents the 95% CI, and the black dotted line represents an OR value of 1.

TSMR analysis based on 129 tumor-associated phenotypes revealed that 13 phenotypes exhibited a potential causal relationship with metformin (*p*-value of IVW < 0.05; Figure 1; Supplementary Table S2). Notably, the OR values between metformin and the 13 tumor-associated phenotypes were < 1, suggesting that metformin may reduce the risk of tumor development. Metformin exerted a protective effect on HER-positive malignant neoplasm of the breast (IVW OR: 0.054, 95% CI: 0.006–0.515, *p*-value: 0.011, P FDR: 0.305), brain meningioma (IVW OR: 0.00, 95% CI: 0.00–0.049, *p*-value: 0.002, P FDR: 0.249), malignant neoplasm of oral cavity (IVW OR: 0.00, 95% CI: 0.00–0.19, *p*-value: 0.014, P FDR: 0.305), and cancer of tonsil and base of tongue (IVW OR: 0.00, 95% CI: 0.00–0.105, *p*-value: 0.014, P FDR: 0.305). The MR analysis results visually illustrated the contribution of each IV to the outcome in the forest plot (Supplementary Figure S1). They provided evidence of a relationship between exposure and outcome in the scatter plot (Supplementary Figure S2). Surprisingly, this relationship was significant after FDR correction in malignant neoplasms of the breast (IVW OR: 0.023, 95% CI: 0.003–0.170, *p*-value: 0.000, P FDR: 0.028).

### Sensitivity analysis and heterogeneity testing

Leave-one-out sensitivity analysis was used to evaluate the effect size of the remaining IVs on the outcome by eliminating individual SNPs individually. The results indicated that these SNPs contributed almost equally to the outcome, and no single SNP dominated the results of the MR analysis (Supplementary Figure S3). Additionally, the heterogeneity test did not detect obvious heterogeneity (Supplementary Table S3), further supported by the funnel plot results, demonstrating that the IVs in the MR analysis were evenly distributed on both sides (Supplementary Figure S4).

### Verification based on testing cohort

**FIGURE 2:**
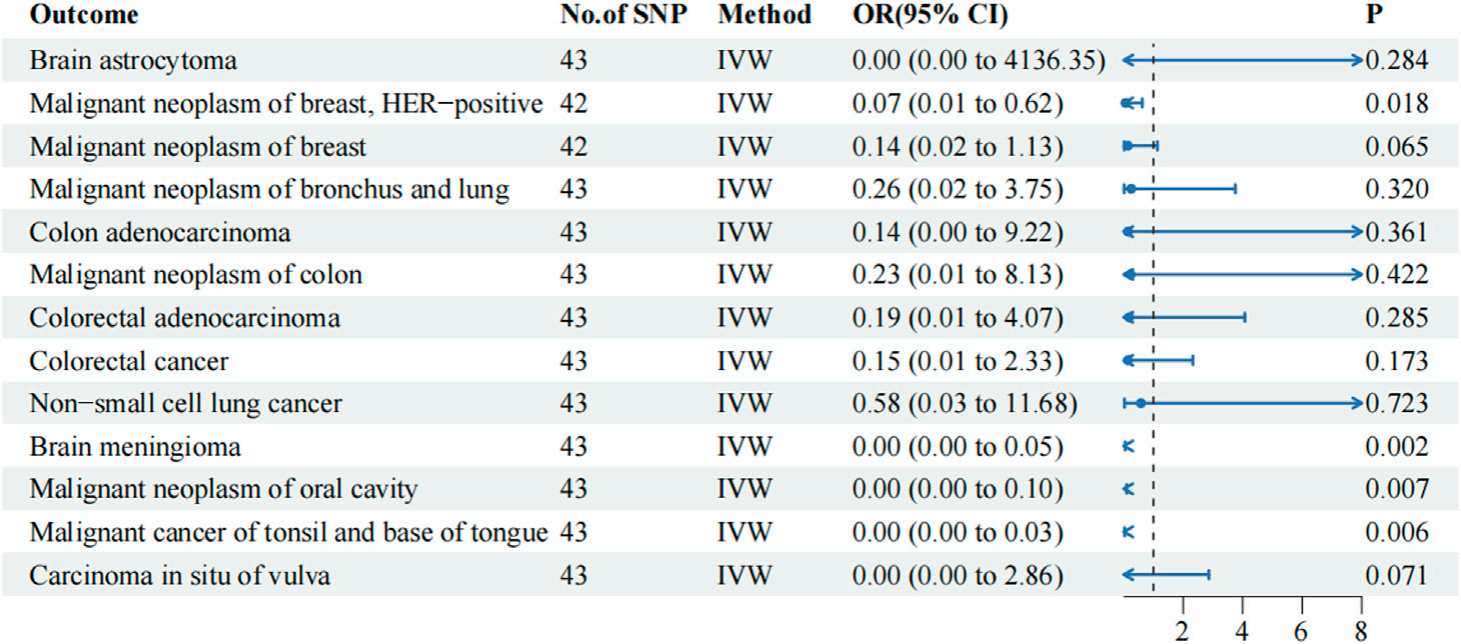
IVW results of metformin against 13 tumor-associated phenotypes in the testing cohort. The blue dots represent the OR value, the blue solid line represents the 95% CI, and the black dotted line represents an OR value of 1.

A TSMR analysis was performed using the same parameters in the testing cohort to verify the reliability of the training cohort results. The findings revealed significant differences among the four malignancy phenotypes in the testing cohort (*p*-value of IVW < 0.05; Figure 2). Similarly, their results suggested that metformin may have a protective effect, with an OR of < 1. In the testing cohort, metformin exhibited a protective effect against HER-positive malignant neoplasms of the breast (IVW OR: 0.065, 95% CI: 0.007–0.625, *p*-value: 0.018, P FDR: 0.577), brain meningioma (IVW OR: 0.000, 95% CI: 0.000–0.051, *p*-value: 0.002, P FDR: 0.194), malignant neoplasm of the oral cavity (IVW OR: 0.000, 95% CI: 0.000–0.099, *p*-value: 0.007, P FDR: 0.289), and cancer of the tonsil and base of the tongue (IVW OR: 0.000, 95% CI: 0.000–0.035, *p*-value: 0.006, P FDR: 0.289). However, similar to the training cohort, no significant difference was observed after the FDR correction. Metformin did not have a causal association with malignant neoplasm of the breast (IVW OR: 0.142, 95% CI: 0.018–1.133, *p*-value: 0.065, P FDR: 0.588) in the testing cohort.

### Meta-analysis based on IVW method of training cohort and testing cohort

**FIGURE 3:**
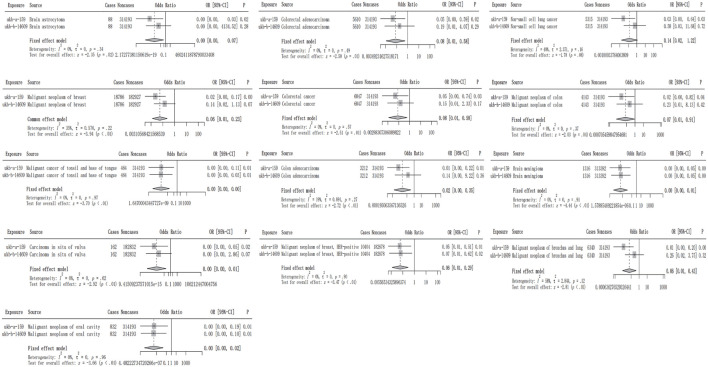
The meta-analysis integrated the IVW results of metformin against 13 tumor-associated phenotypes in the training and testing cohorts. The gray rectangle represents the OR value, the black line segment represents the 95% CI, and the black dotted line represents the OR value after integrating the effect size.

To further enhance the level of evidence for a causal relationship, a meta-analysis was conducted by integrating the IVW results from the training and testing cohorts. All combinations exhibited I^2^ ≤ 50% and *p*-value ≥ 0.05; therefore, a fixed effects model was selected to combine the IVW results. Although only five types of malignancies were verified in the testing cohort, surprisingly, in the meta-analysis, all 12 results were significant (Figure 3). This suggests that metformin may influence the risk of tumor development, although this relationship may not be causal.

### Mediational MR analysis

**FIGURE 4:**
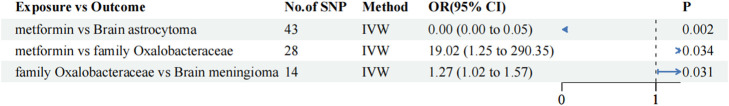
Mediating MR analysis among metformin, the Oxalobacteraceae family, and brain meningioma. The blue dots represent the OR value, the blue solid line represents the 95% CI, and the black dotted line represents an OR value of 1.

Despite our efforts to explore the potential mechanism by which metformin affects tumor risk using mediated MR analysis, no variables exhibiting significant differences in pairwise TSMR were identified in the field of lipid metabolism/immune cells. In intestinal flora, we identified that the family Oxalobacteraceae may mediate the effect of metformin on the risk of meningioma. The TSMR analysis revealed significant differences among the three groups. Metformin versus brain meningioma (IVW OR: 0.000, 95% CI: 0.000–0.051, *p*-value: 0.002), metformin versus family Oxalobacteraceae (IVW OR: 19.016, 95% CI: 1.245–290.35, *p*-value: 0.034), family Oxalobacteraceae versus brain meningioma (IVW OR: 1.266, 95% CI: 1.022–1.569, *p*-value: 0.031) (Figure 4). However, the family Oxalobacteraceae seemed to be a mediating factor in attenuating the anti-tumor effect of metformin, as it accounted for −8.9% of the total effect. Although all three were found to be significantly different, this significance no longer existed in the Sobel test (*p*-value = 0.131).

## Discussion

Although metformin has demonstrated potential anti-tumor activity, this relationship does not seem stable, as numerous studies have reported inconsistent results. This study investigated the causal relationship between the two through MR-PheWAS analysis associated with tumors. Our study identified 13 phenotypes in a training cohort in which metformin demonstrated a potential causal relationship. Four phenotypes were verified in the testing cohort, including four different tumor types: malignant neoplasm of the breast (HER-positive), brain meningioma, malignant neoplasm of the oral cavity, and cancer of the tonsil and base of the tongue. Several observational studies in the Chinese population have reported that metformin has a protective effect against hepatocellular carcinoma (Tseng, 2018a), gastric cancer (Tseng, 2016a), and nasopharyngeal cancer (Tseng, 2018b). However, this result was not reflected in the present study and may have been affected by the population from different sources. Although no significant difference was observed between them after FDR correction, it is noteworthy that 12 phenotypes retained their potential causal relationship with metformin after the integration of findings through a meta-analysis. These results indicate that metformin may have potential anti-tumor effects.

In a population-based observational study of 476,282 Asian women with newly diagnosed T2DM, metformin use was found to significantly reduce the incidence of breast cancer (Tseng, 2014). In a retrospective study, the MD Anderson Cancer Center included 1,448 TNBC patients who received adjuvant chemotherapy. It conducted survival analysis by dividing the patients into groups based on their diabetes status and metformin use. Although metformin can potentially reduce the risk of distant metastasis, it does not affect OS (Bayraktar et al., 2012). Another retrospective analysis revealed that the 5-year OS of breast cancer patients with diabetes who used metformin was significantly longer than that of patients without metformin and diabetes-free breast cancer (Hou et al., 2013). One of the differences between these two studies was that they were inconsistent in their consideration of pathological classification, which suggests that molecular classification may be necessary to determine whether metformin exerts a better anti-tumor effect.

Although numerous studies have explored the relationship between metformin and tumors, there seems to be a scarcity of research on benign brain tumors such as meningiomas. A retrospective cohort study investigated the relationship between metformin and benign brain tumors (BBT) and revealed that metformin use reduced the risk of BBT, especially meningiomas, in patients with T2DM (Tseng, 2021). Clinically, there are significantly more female patients with meningioma than male patients, with the ratio ranging from 2 to 3:1 (Claus et al., 2005; Cao et al., 2022). This ratio can be as high as 9:1 in spinal meningiomas. Hormones are considered risk factors for meningiomas. A meta-analysis including 1,600 patients with meningiomas revealed a significant association between prior hormone therapy and an increased risk of developing meningioma (RR 1.35, 95% CI 1.2–1.5) (Benson et al., 2015). Metformin has been shown to regulate hormone levels. In one study, metformin reduced androgen and estrogen levels in non-diabetic breast cancer patients (Campagnoli et al., 2013). Notably, women with breast cancer have a moderately increased risk of meningioma, and a similar increase in breast cancer rates is observed in women with a history of meningioma (Custer et al., 2002; Wiemels et al., 2010). Therefore, the potential protective effects of metformin against meningiomas may be closely related to hormonal regulation. A study using UK-based Clinical Practice Research Datalink (CPRD) data revealed no clear relationship between metformin use and meningioma risk (Seliger et al., 2017). However, after adjusting for the duration of diabetes and HAbc1, short-term use of metformin seemed to have a potential protective effect (OR: 0.84, 95% CI: 0.43–1.64; *p*-value for trend = 0.059).

In addition to meningiomas, we observed that metformin potentially exhibited anti-tumor effects against malignant neoplasms of the oral cavity, tonsil, and tongue base. Intriguingly, most of the four positive phenotypes in this study were located in the head and neck regions. In oral squamous cell carcinoma (OSCC), the transcription factor LSF (Late SV40 Factor) binds to the promoter region of Aurora-A to induce carcinogenesis. Metformin inhibits the LSF/Aurora-A signaling pathway *in vitro* and in xenograft models, thereby mitigating tumor risk (Chen et al., 2017). However, in a 4-NQO rat oral cancer model, using low and high doses of metformin had no significant effect on the invasion score of OSCC and could not change the occurrence of precancerous lesions (Thompson et al., 2017). An observational study in Taiwan identified a reduced risk of oral cancer associated with metformin use (Tseng, 2016b). The patients’ pathology was significantly alleviated in a single-arm IIa clinical trial. Metformin was administered to 23 patients with oral precancerous lesions without T2DM, which is closely related to the inhibition of the mTOR signaling pathway by metformin (Gutkind et al., 2021). In the study of metformin and tongue cancer, only one study showed that doxorubicin combined with metformin can inhibit the growth, invasion, and migration of SSC-15 cells and promote SSC-15 cell apoptosis (Zhang, 2019).

Although metformin has been reported to have multiple biological functions and anti-tumor effects, this mechanism has rarely been clinically studied. This study explored the causal relationship between metformin and four positive tumor phenotypes. Unfortunately, TSMR analysis does not identify any factors that differ significantly among the three in lipid metabolism and immune cells. Notably, metformin, the family Oxalobacteraceae, and brain meningiomas have significant differences simultaneously within intestinal flora. The Oxalobacteraceae family appears to be a blocking factor that causes metformin to reduce the risk of brain meningioma, accounting for −8.9% of the total effect. The Oxalobacteraceae family is closely related to the risk of T2DM. One study used a high-fat and high-fructose diet to feed mice as a T2DM animal model. The findings revealed that the intestinal family Oxalobacteraceae significantly increased compared with the control group (Costabile et al., 2022). Another MR analysis demonstrated that an increased presence of the family Oxalobacteraceae was associated with the risk of T2DM (Zeng et al., 2024). Studies have shown that the therapeutic effects and side effects of metformin are closely related to changes in intestinal flora (Forslund et al., 2017; Wu et al., 2017). Although the *p*-value of the Sobel test ultimately lacked significance, it is worth noting that modulating the intestinal flora to alleviate the adverse effects of metformin was essential for improving its efficacy in treating tumors.

Our study identified the potential of metformin in reducing the risk of four specific types of tumors. Furthermore, we observed that the Oxalobacteraceae family may attenuate this effect in meningiomas. This suggests that the side effects of metformin can further improve its ability to reduce the risk of cancer. Variations in the sample size and population structure were observed despite all populations being European owing to different database sources. Therefore, this study established a training group and a testing group to balance the limitations of a single dataset and bias, further providing relatively reliable results. To further consolidate the reliability of the conclusions, we also integrated these two cohorts for the meta-analysis. Although our study revealed this possibility, there are still some limitations. First, the database and ethnic sources were limited to the European population, which is not conducive to generalizing the conclusions to the Asian population or even wider areas. Second, although MR analysis has become an important method for causal assessment, it is only based on statistical analysis. The actual clinical situation is very complex, and there are many factors to consider; therefore, randomization of large samples is still needed for analysis and verification.

## Conclusion

The present study demonstrated that metformin may reduce the risk of four different malignancies. However, there was no clear causal relationship between metformin use alone and cancer risk. Large randomized controlled trials are required to validate these findings.

## Data Availability

The original contributions presented in the study are included in the article/Supplementary Material, further inquiries can be directed to the corresponding author.

## Supplementary material

SUPPLEMENTARY FIGURE S1 Forest plot of the MR effect size of metformin against 13 tumor-associated phenotypes in the training cohort. The black dots represent the OR value of each SNP, the black line segment represents the 95% CI of each SNP, the red dots represent the OR value calculated by MR-Egger and IVW, and the red line segment represents the final 95% CI calculated by MR-Egger and IVW.

SUPPLEMENTARY FIGURE S2 Scatter plot of the effect distribution of all SNPs in the training cohort. The black dots represent the effects of each SNP, and the trend lines of different colors represent different statistical methods.

SUPPLEMENTARY FIGURE S3 Leave-one-out sensitivity analysis revealed that no single SNP significantly affected the outcome in the training cohort. The black dots represent the beta value of the remaining SNP after each SNP was eliminated, the black line segment represents the 95% CI, the red dot represents the beta value of all SNPs integrated by leave-one-out sensitivity analysis, and the red line segment represents the 95% CI.

SUPPLEMENTARY FIGURE S4 Funnel plot of the distribution of all SNPs in the training cohort.
