# Supplementary material for: Bridging the gap: exploring the causal relationship between metformin and tumors
Source: Front Genet. 2024 Jun 19;15:1397390. doi: 10.3389/fgene.2024.1397390 (PMC11220117; doi:10.3389/fgene.2024.1397390)
Supplement: Supplementary file 1 [file DataSheet7.PDF]

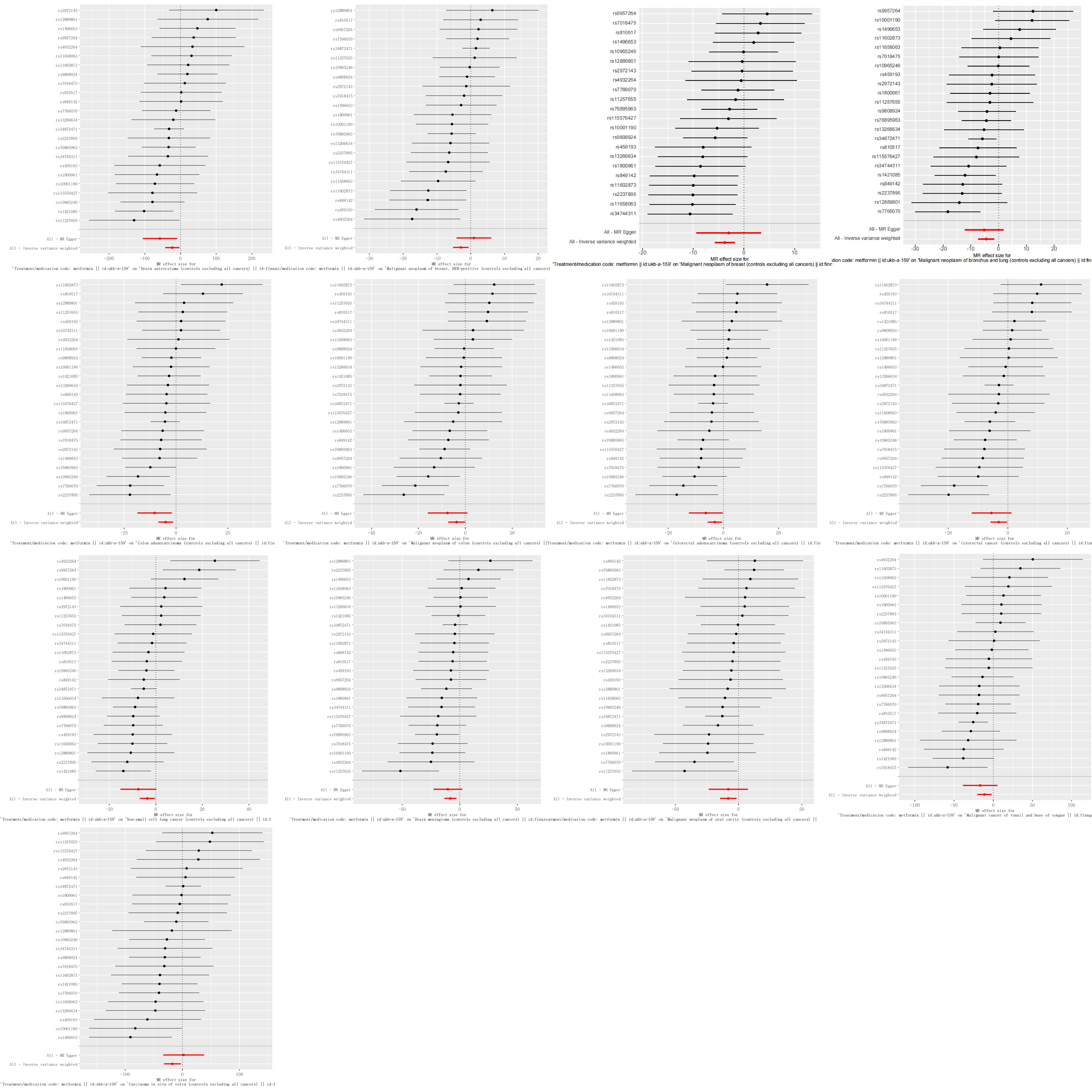

All - MR Egger
All - Inverse variance weighted
MR effect size for
'Treatment/medication code: metformin || id:ukb-a-159' on 'Brain astrocytoma (controls excluding all cancers)' || id:finn
nt/medication code: metformin || id:ukb-a-159' on 'Malignant neoplasm of breast, HER-positive (controls excluding all cancers)
'Treatment/medication code: metformin || id:ukb-a-159' on 'Malignant neoplasm of breast (controls excluding all cancers)' || id:finn
tion code: metformin || id:ukb-a-159' on 'Malignant neoplasm of bronchus and lung (controls excluding all cancers)' || id:fi
'Treatment/medication code: metformin || id:ukb-a-159' on 'Colon adenocarcinoma (controls excluding all cancers)' || id:fin
'Treatment/medication code: metformin || id:ukb-a-159' on 'Malignant neoplasm of colon (controls excluding all cancers)' ||
Treatment/medication code: metformin || id:ukb-a-159' on 'Colorectal adenocarcinoma (controls excluding all cancers)' || id:fin
'Treatment/medication code: metformin || id:ukb-a-159' on 'Colorectal cancer (controls excluding all cancers)' || id:fin
'Treatment/medication code: metformin || id:ukb-a-159' on 'Non-small cell lung cancer (controls excluding all cancers)' || id:f
'Treatment/medication code: metformin || id:ukb-a-159' on 'Brain meningioma (controls excluding all cancers)' || id:finn
reatment/medication code: metformin || id:ukb-a-159' on 'Malignant neoplasm of oral cavity (controls excluding all cancers)' ||
'Treatment/medication code: metformin || id:ukb-a-159' on 'Malignant cancer of tonsil and base of tongue' || id:finge
'Treatment/medication code: metformin || id:ukb-a-159' on 'Carcinoma in situ of vulva (controls excluding all cancers)' || id:f
