# Supplementary figures and images for: Bridging the gap: exploring the causal relationship between metformin and tumors
Source: Front Genet. 2024 Jun 19;15:1397390. doi: 10.3389/fgene.2024.1397390 (PMC11220117; doi:10.3389/fgene.2024.1397390)

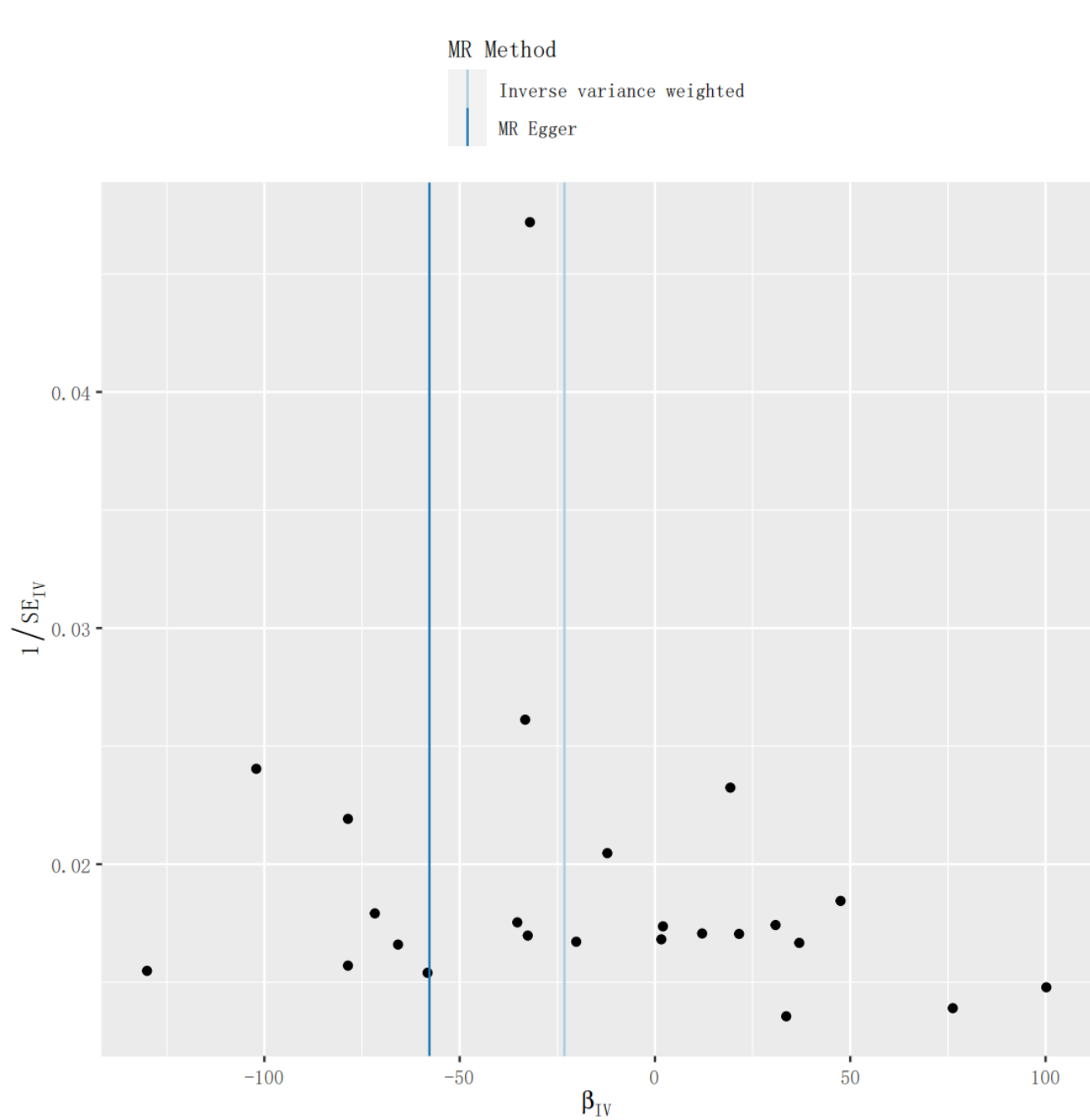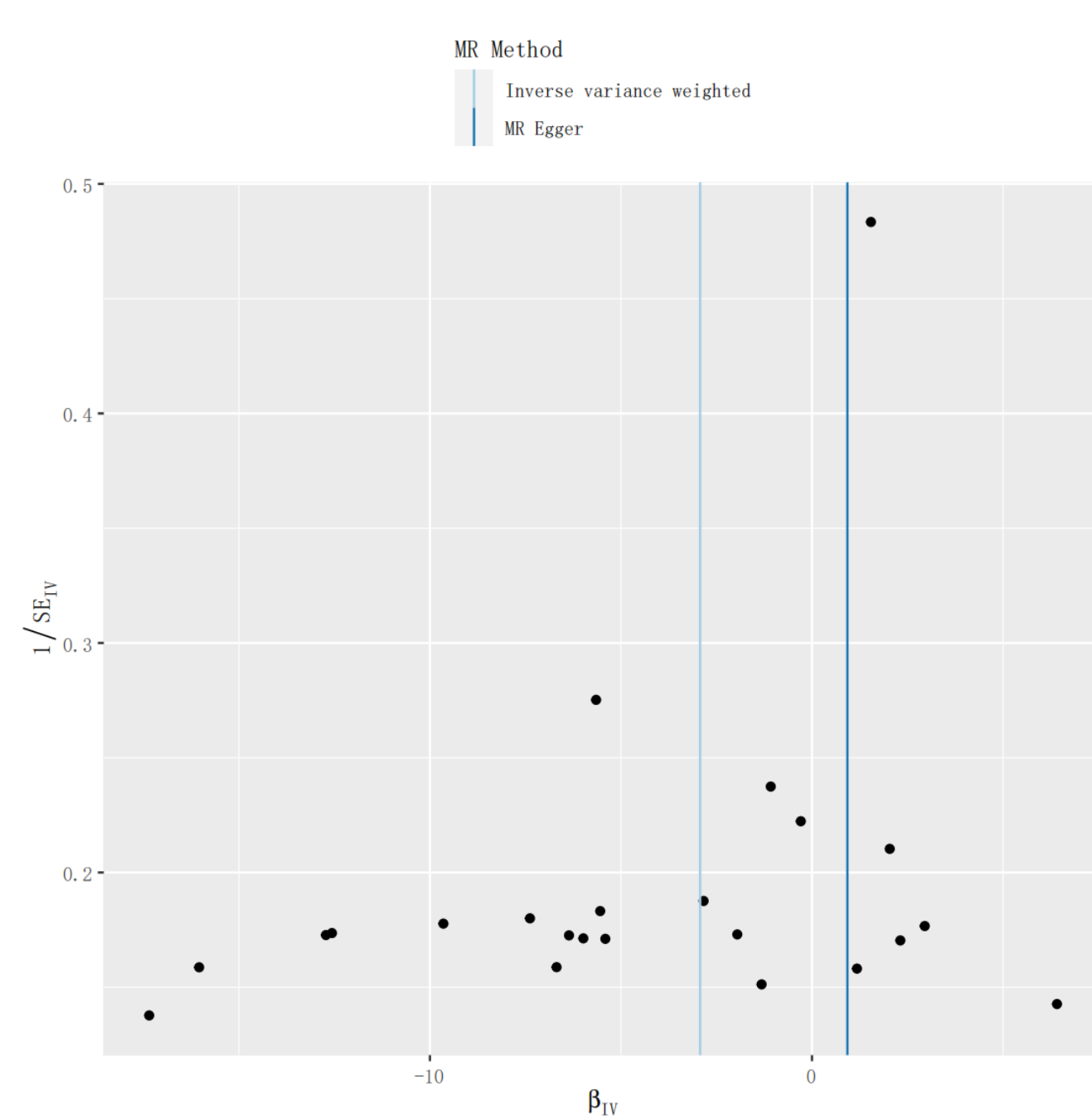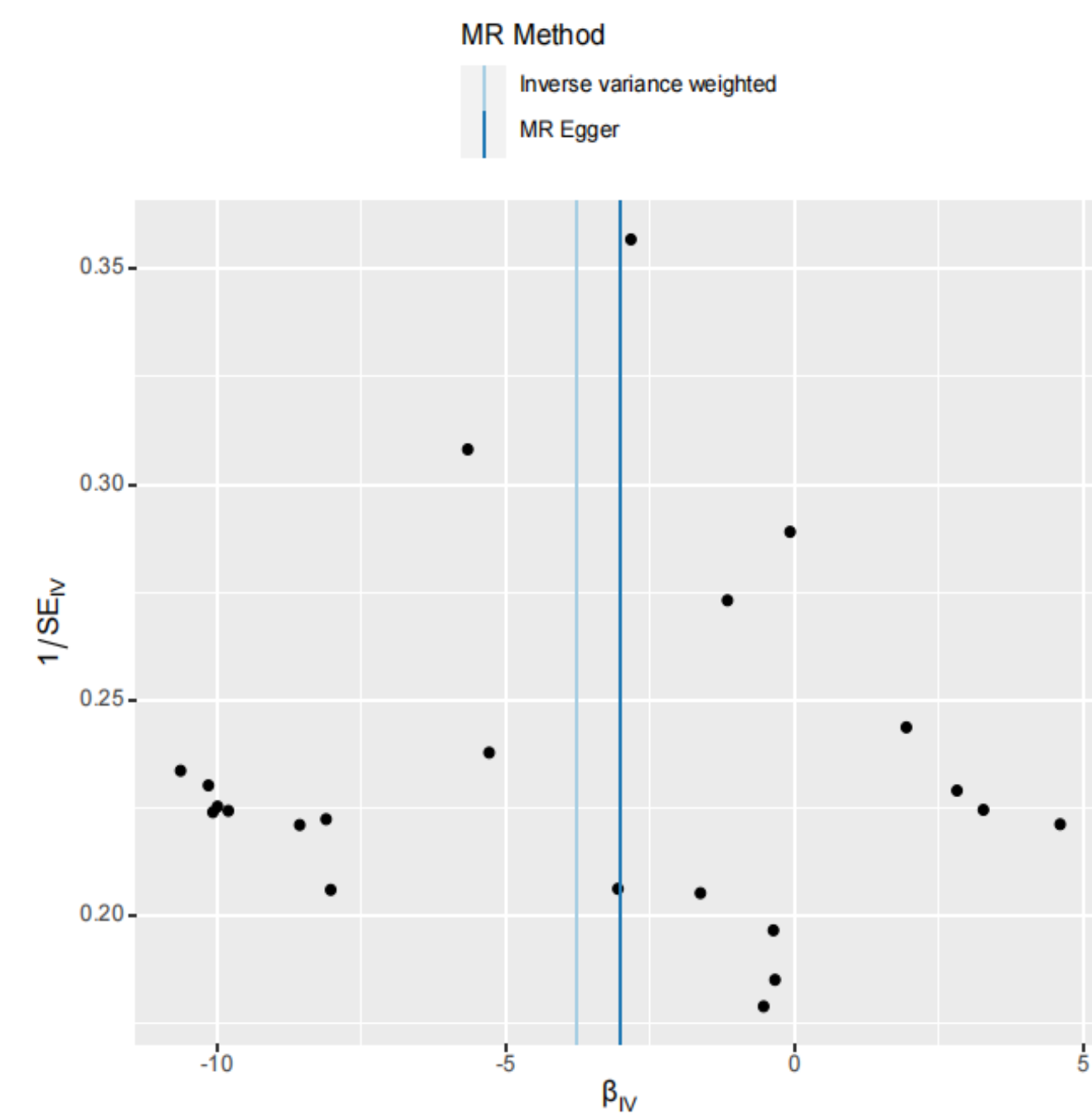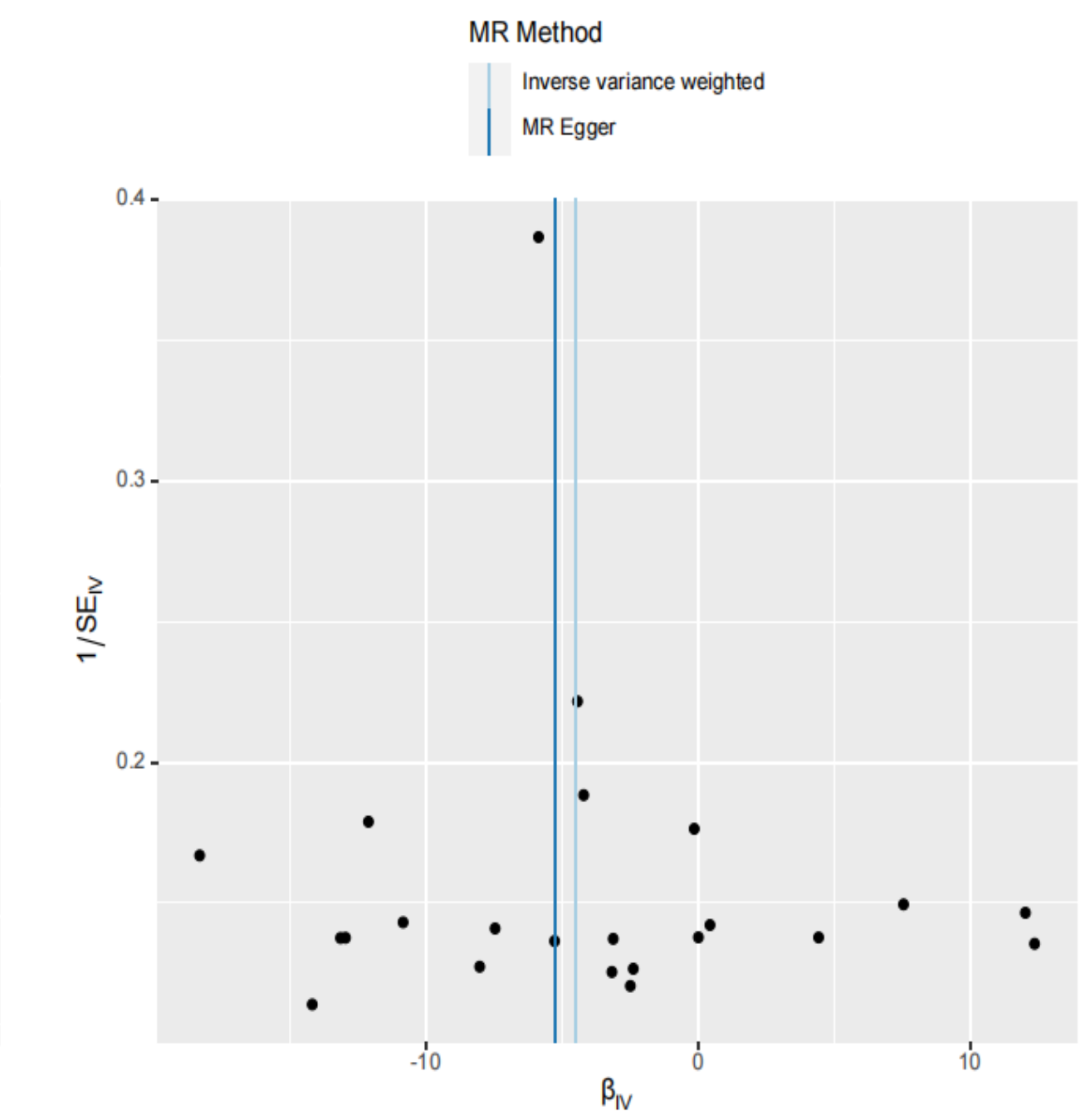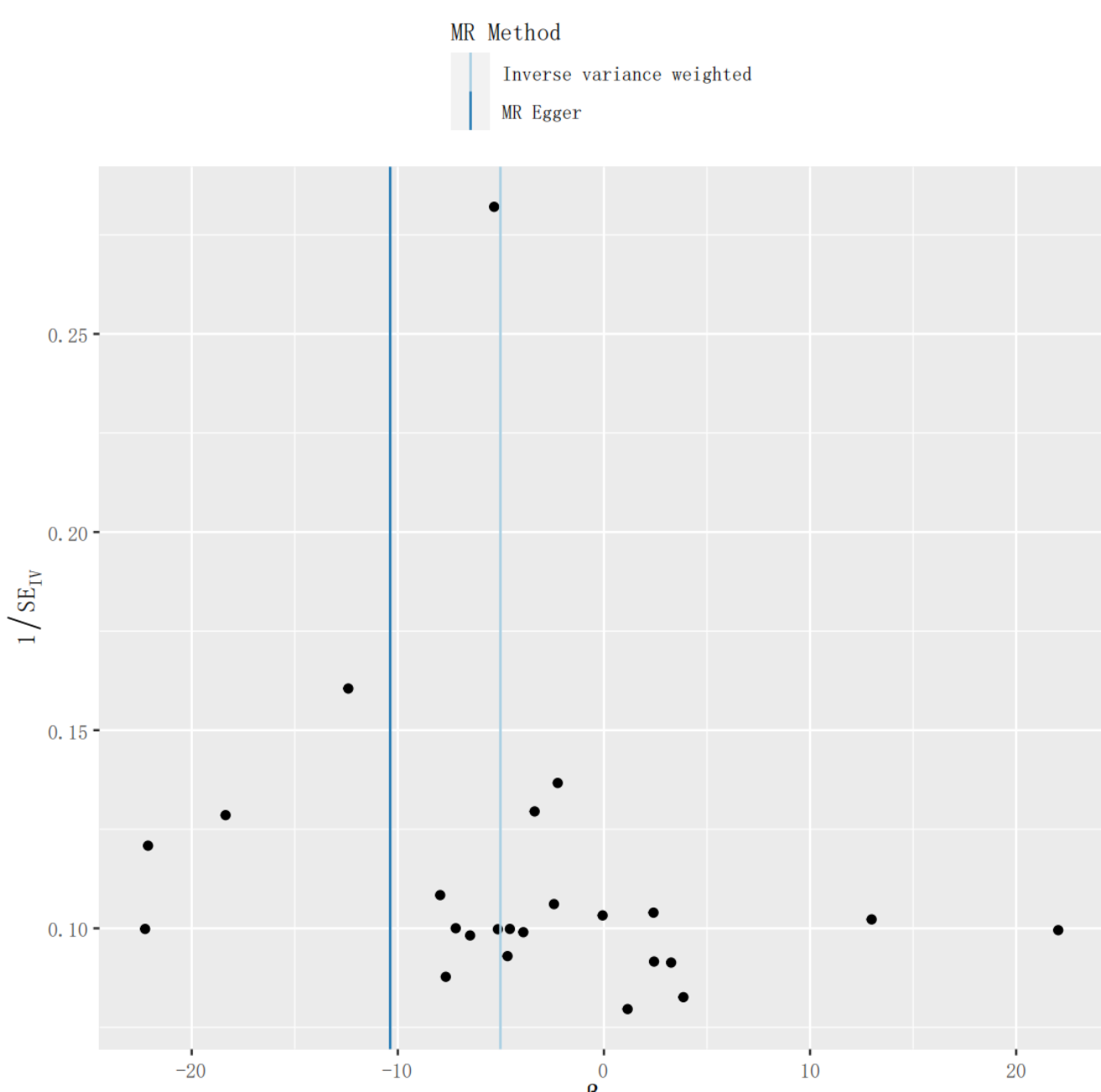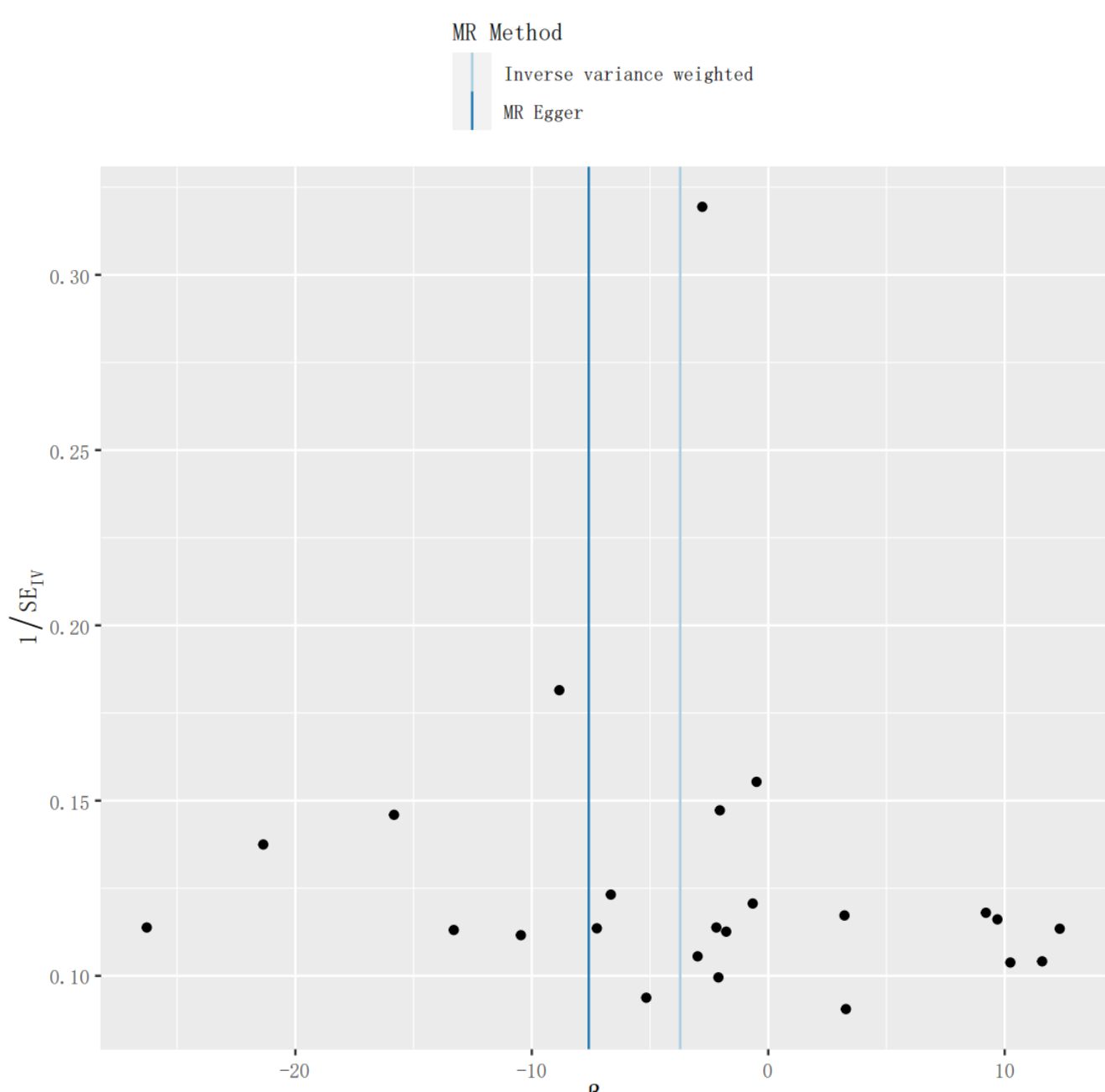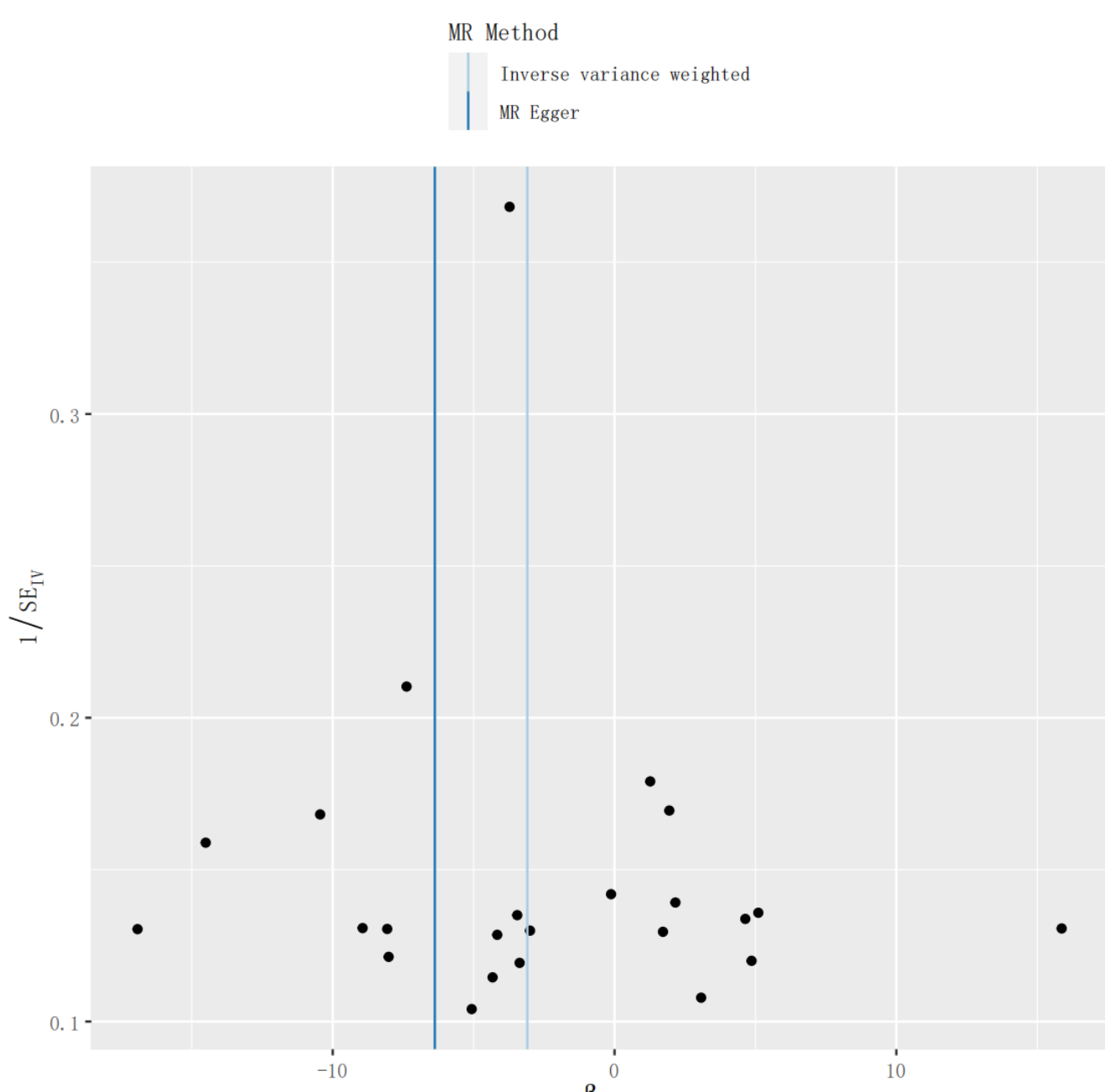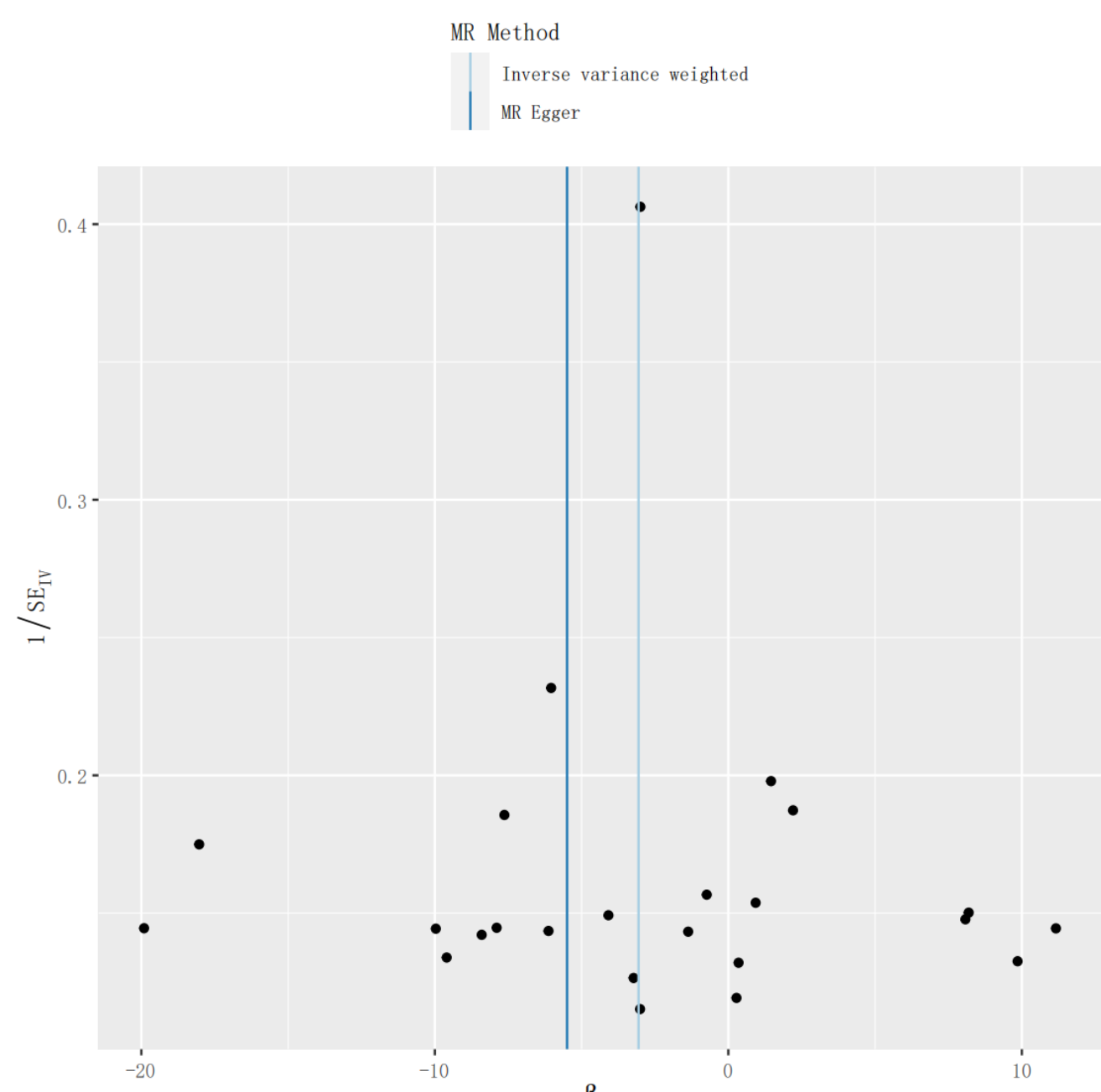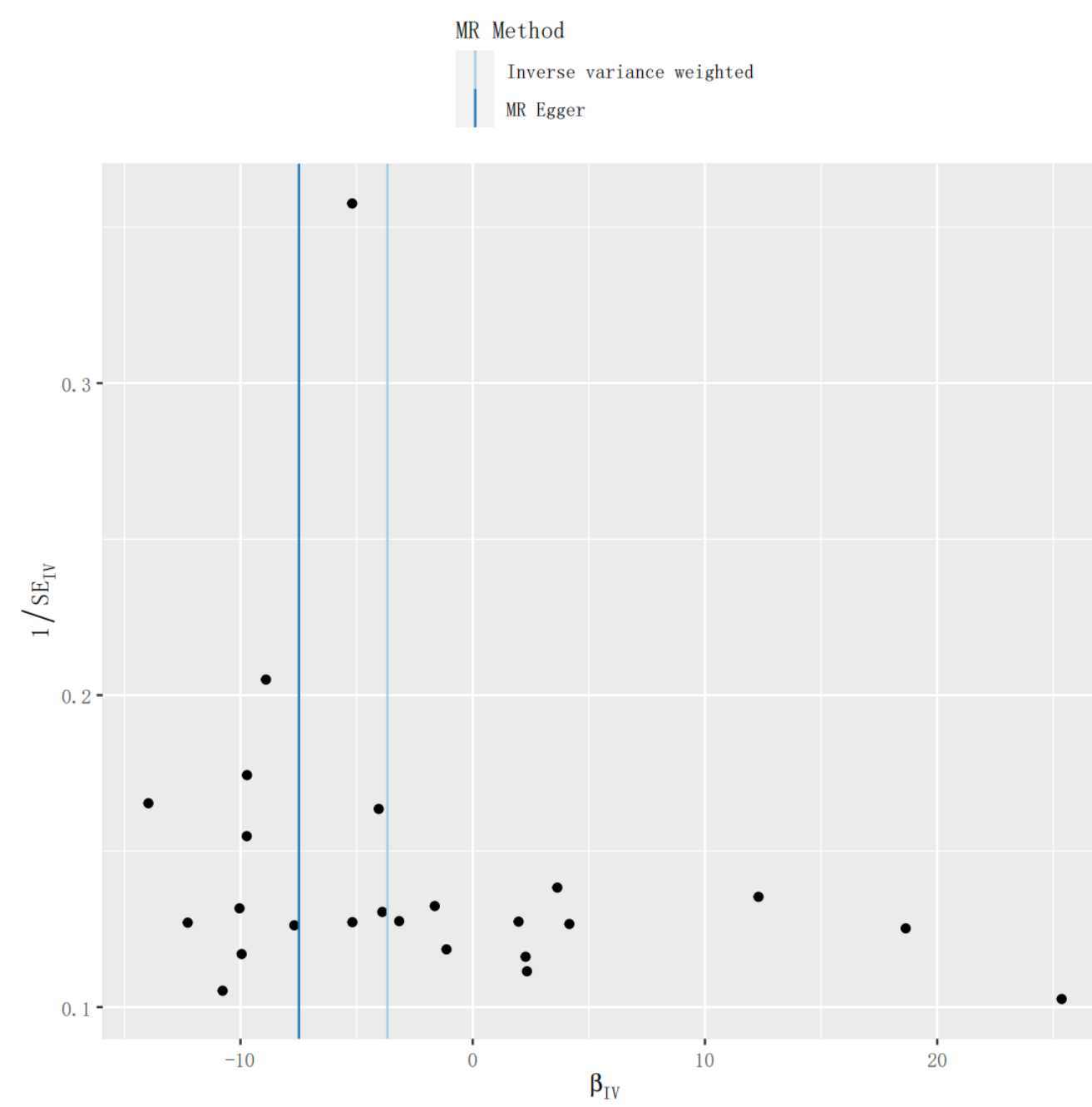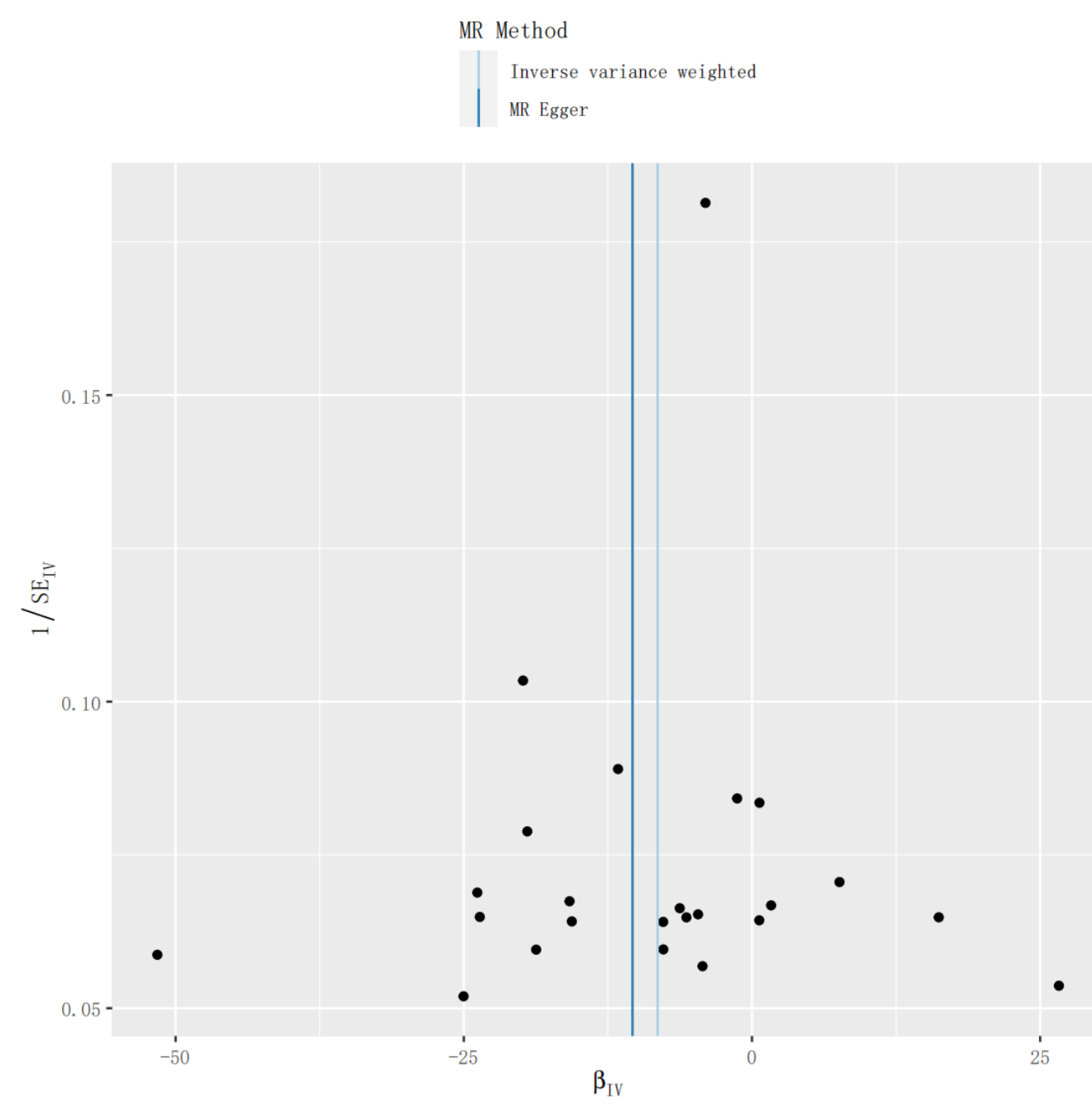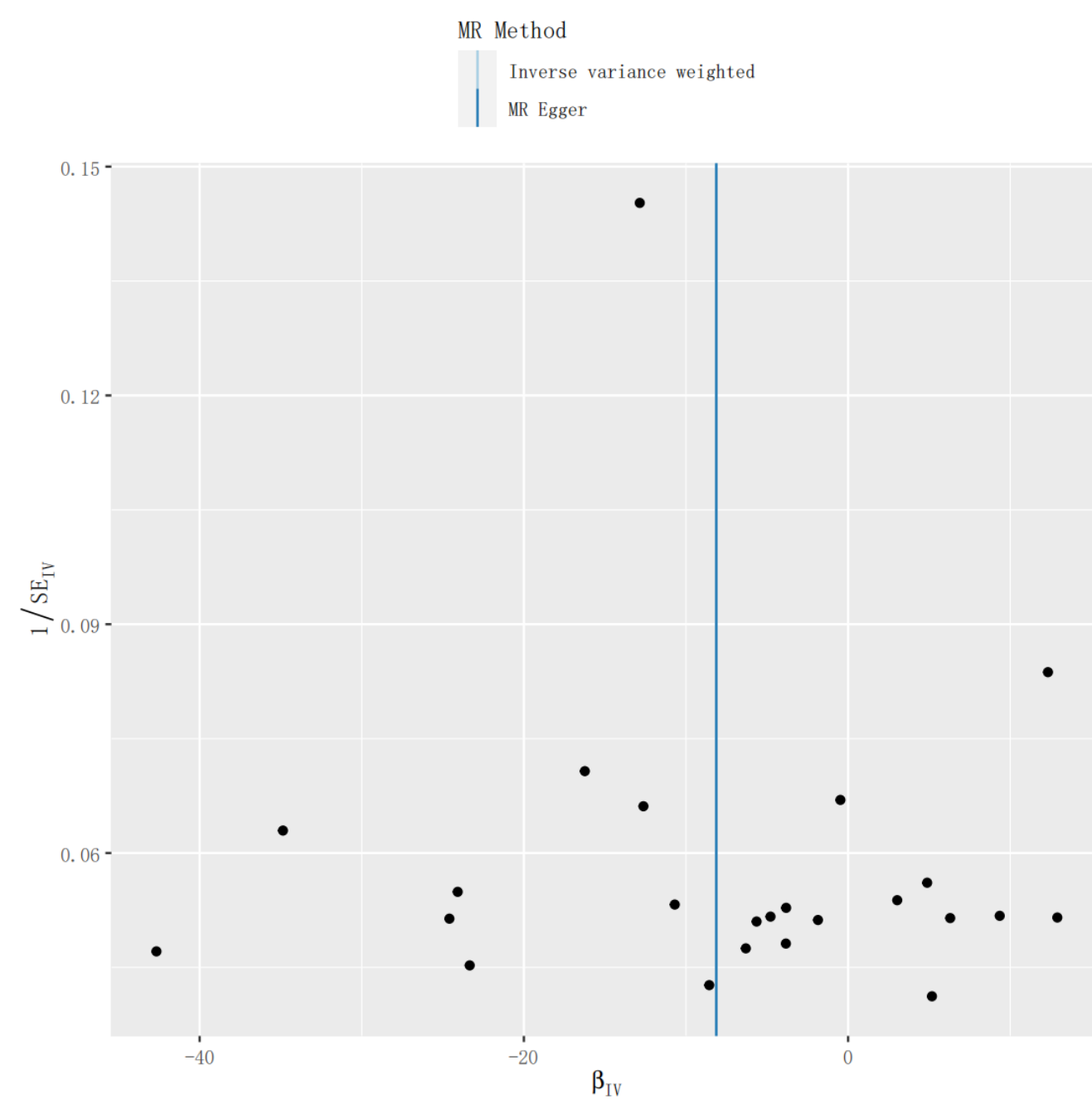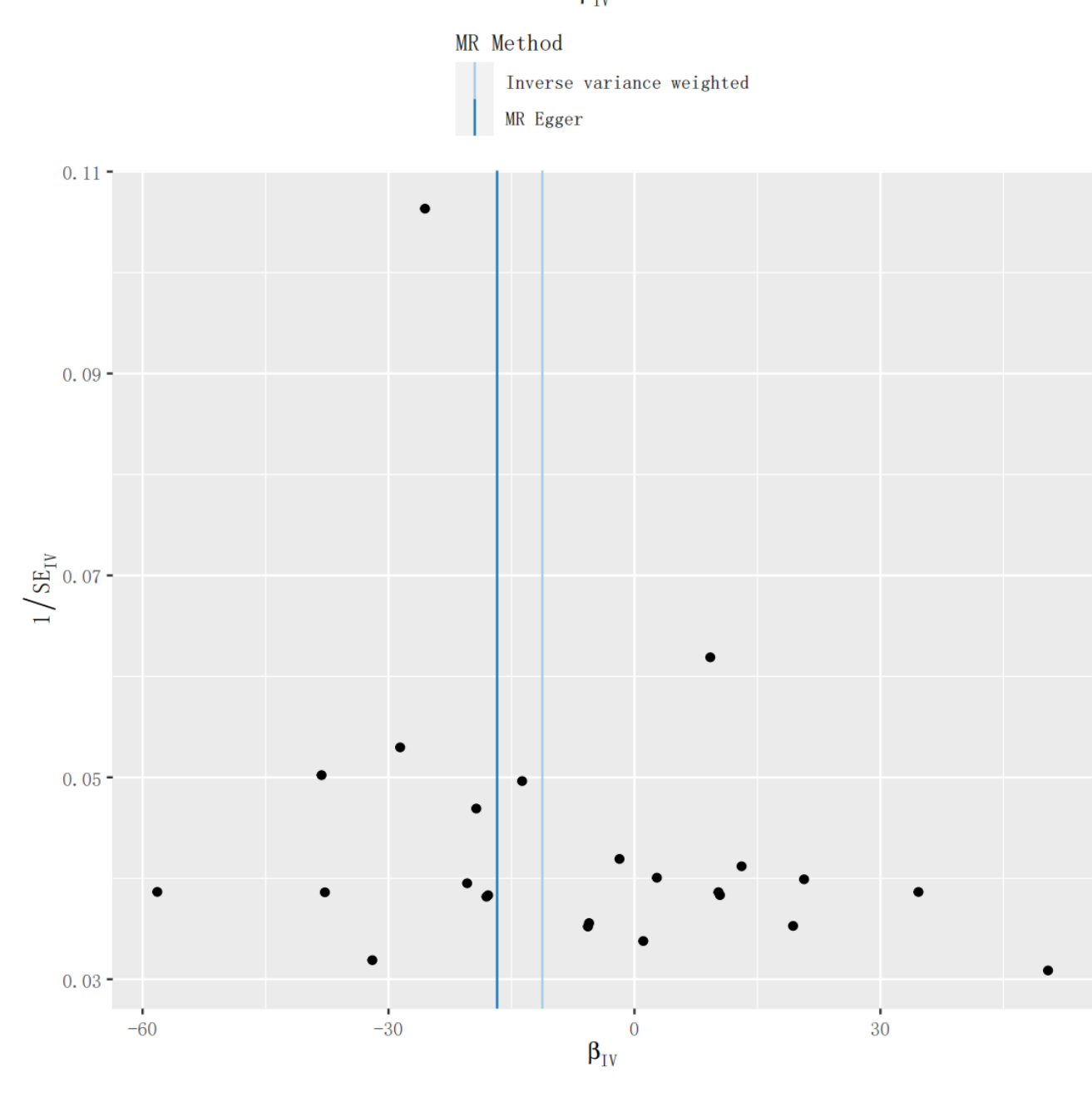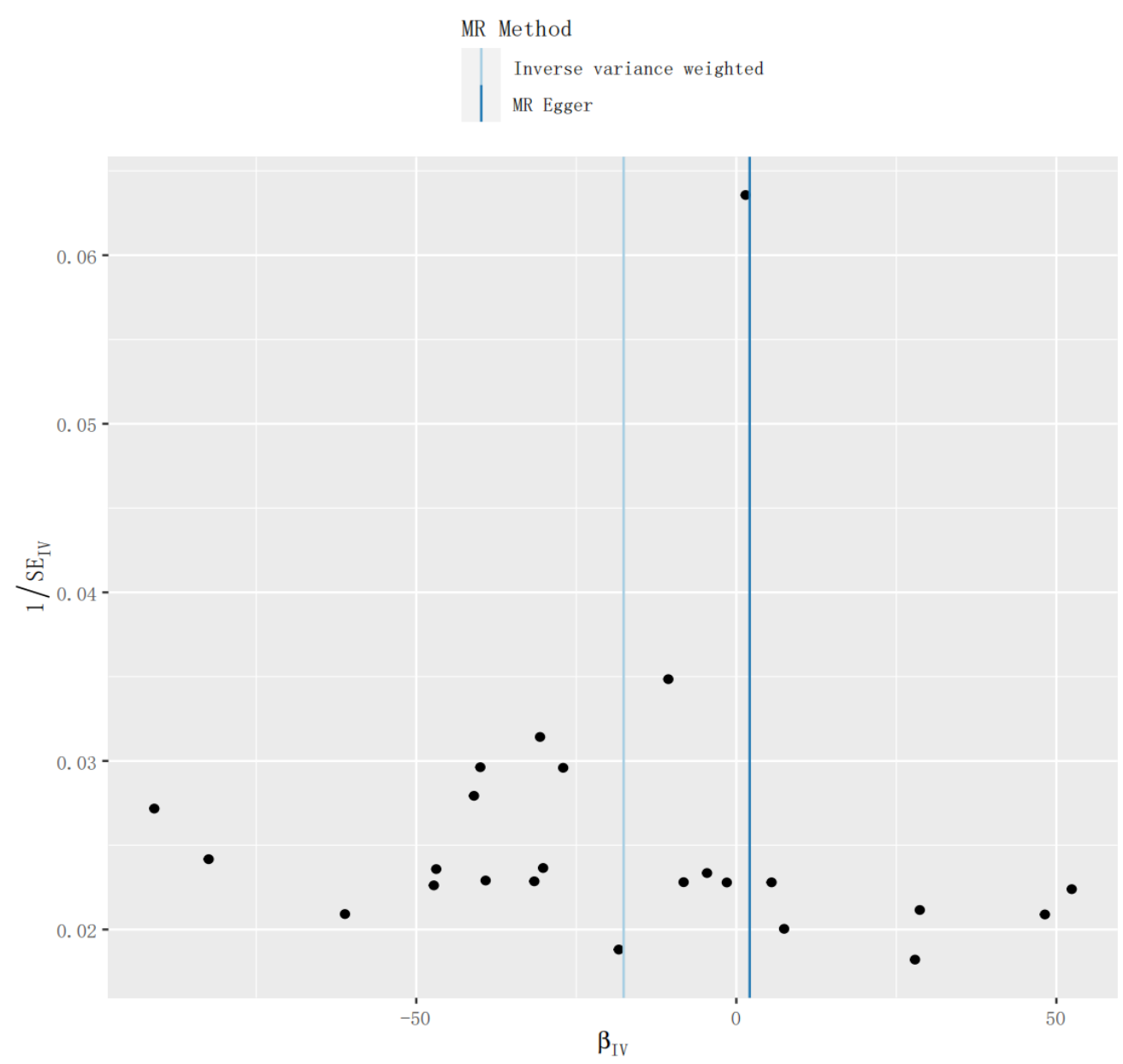

Supplement: Supplementary file 3 [file DataSheet6.PDF]

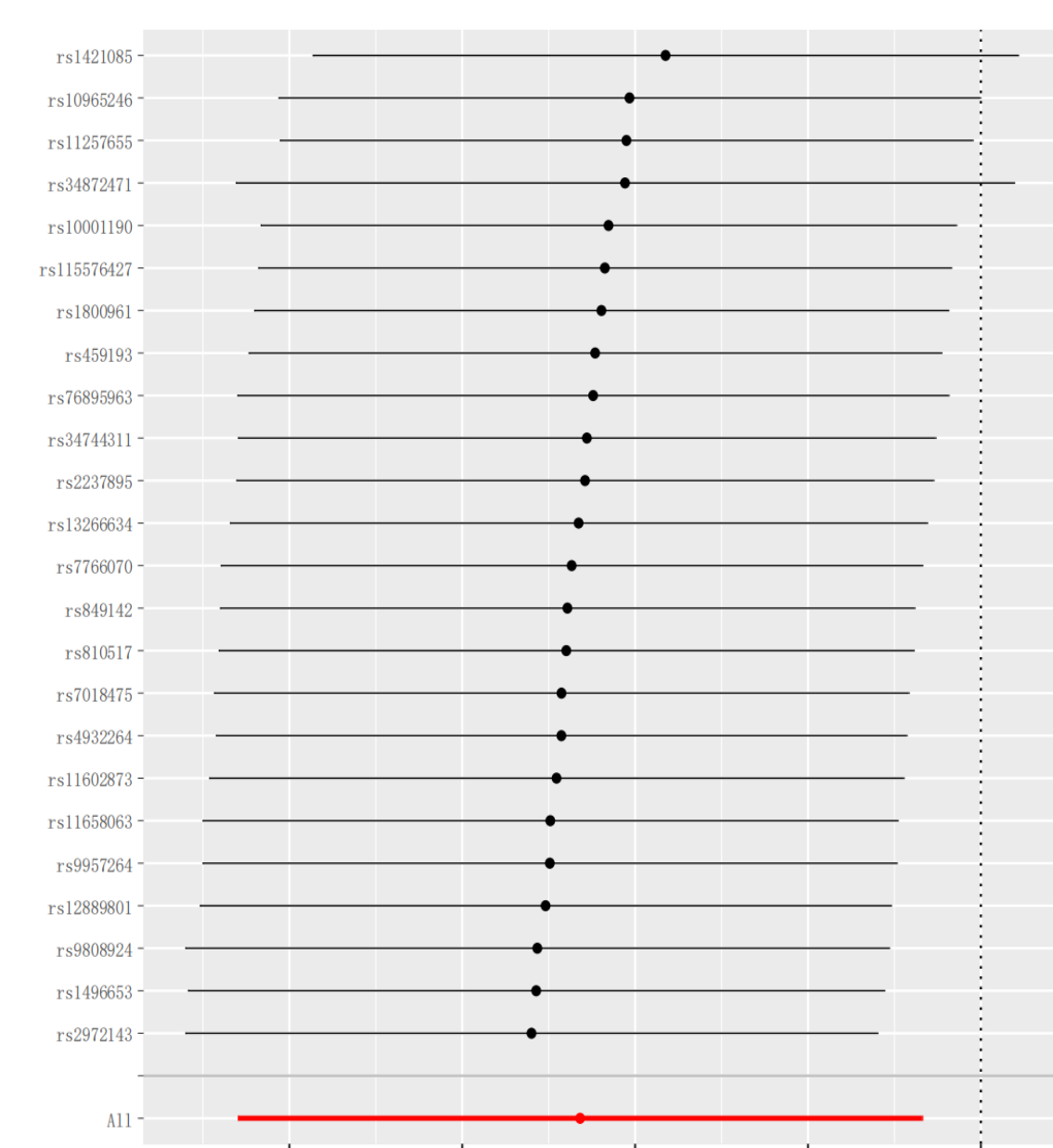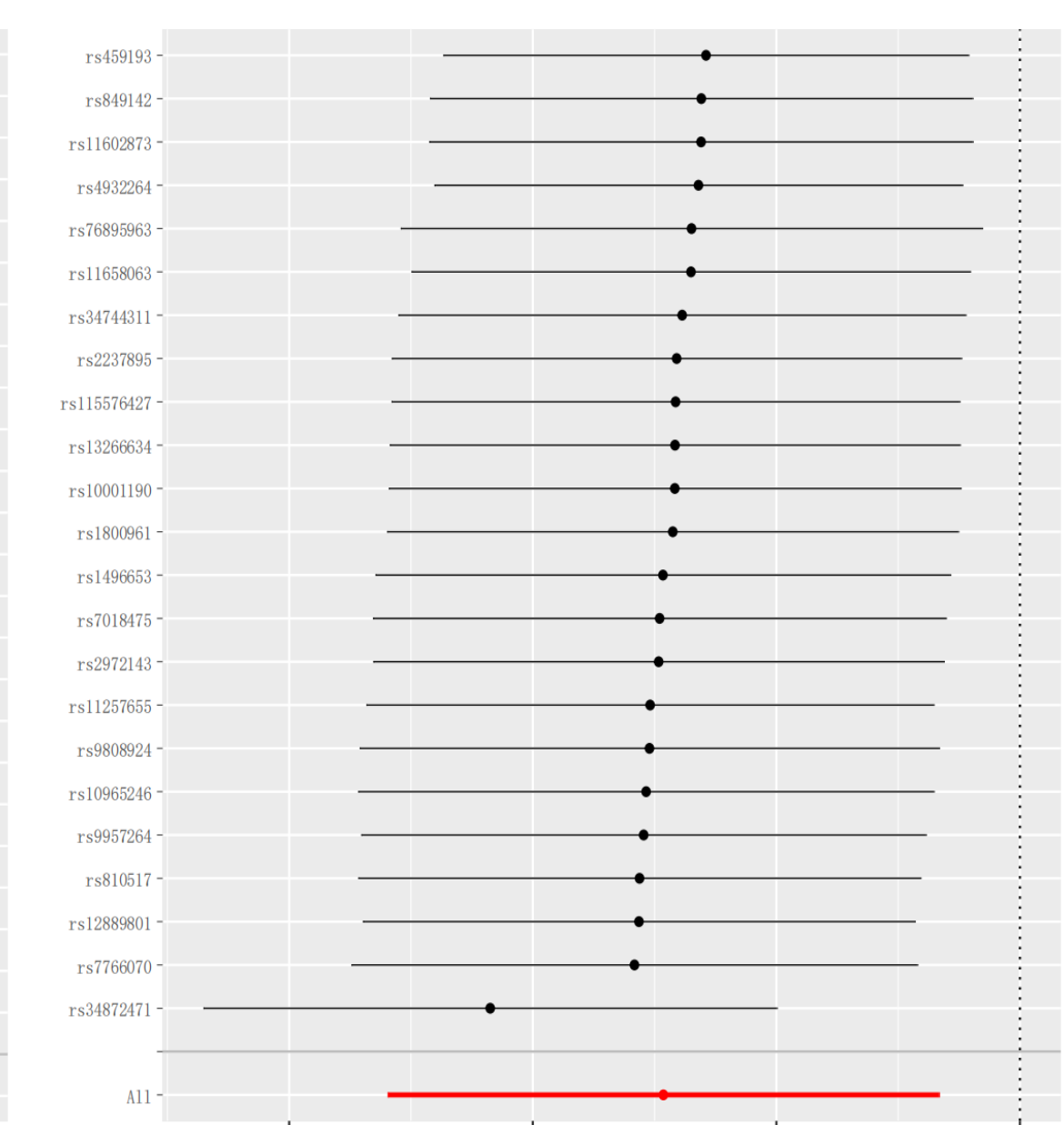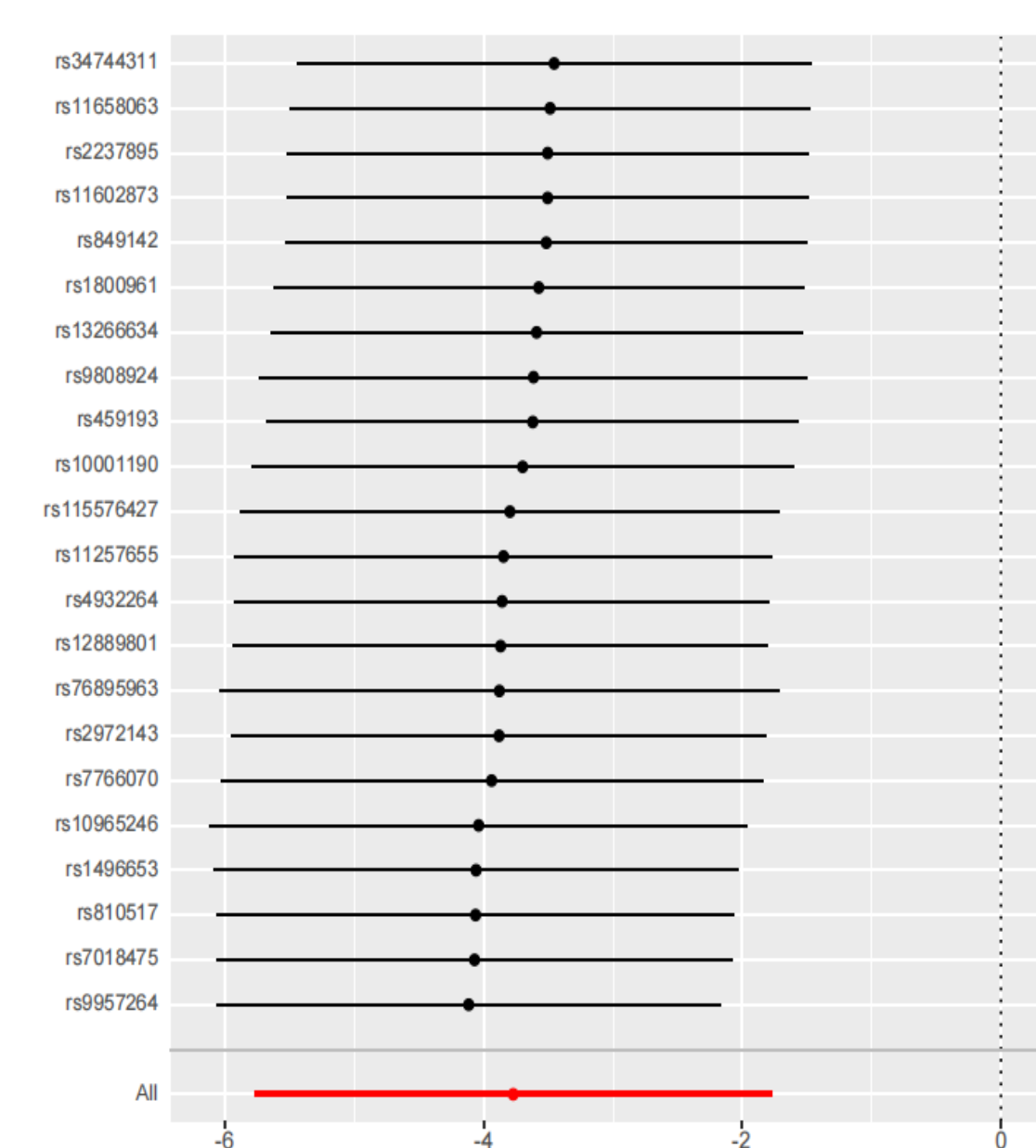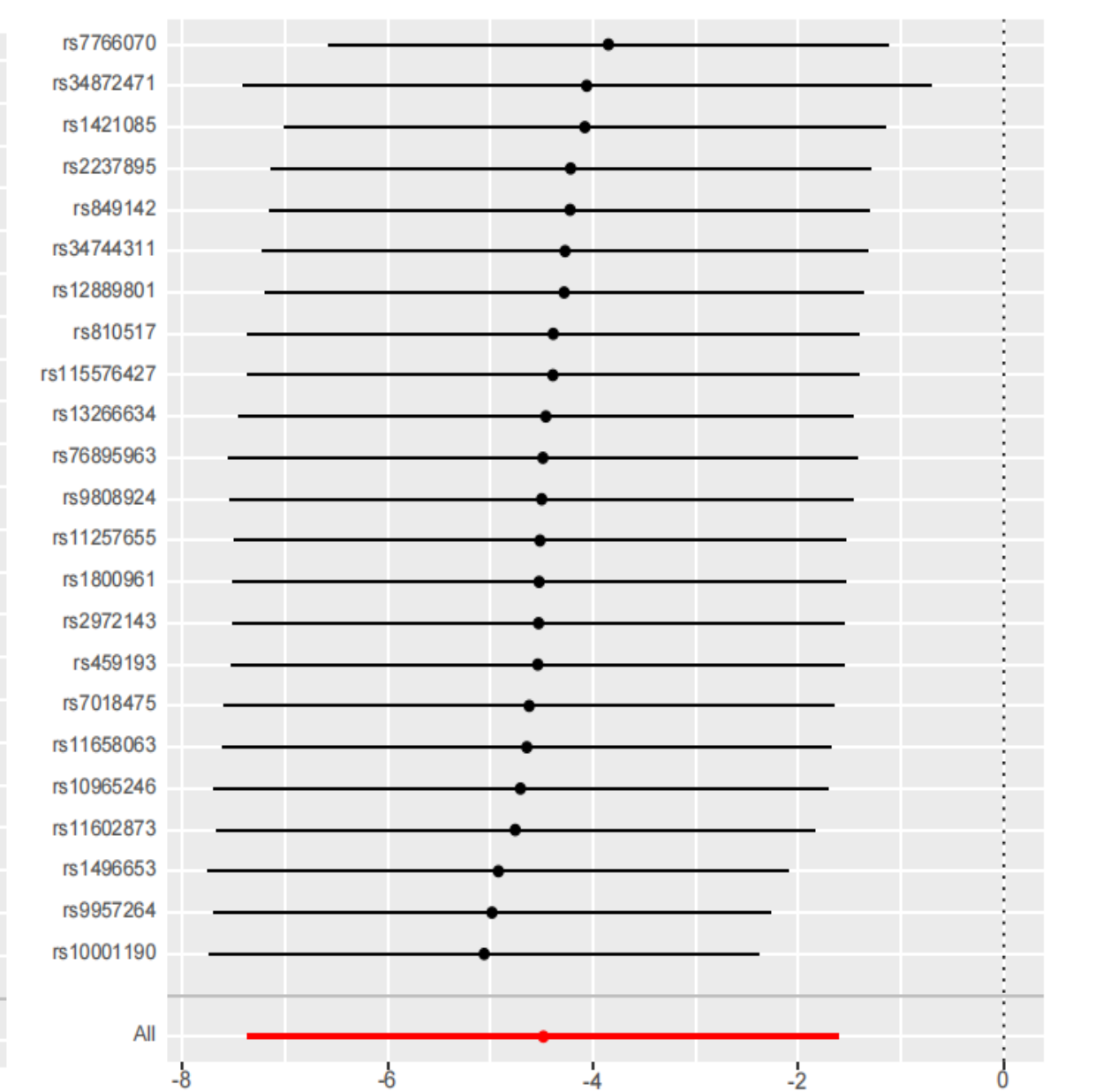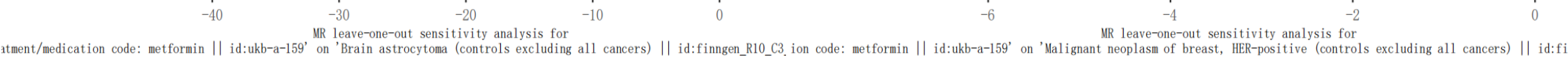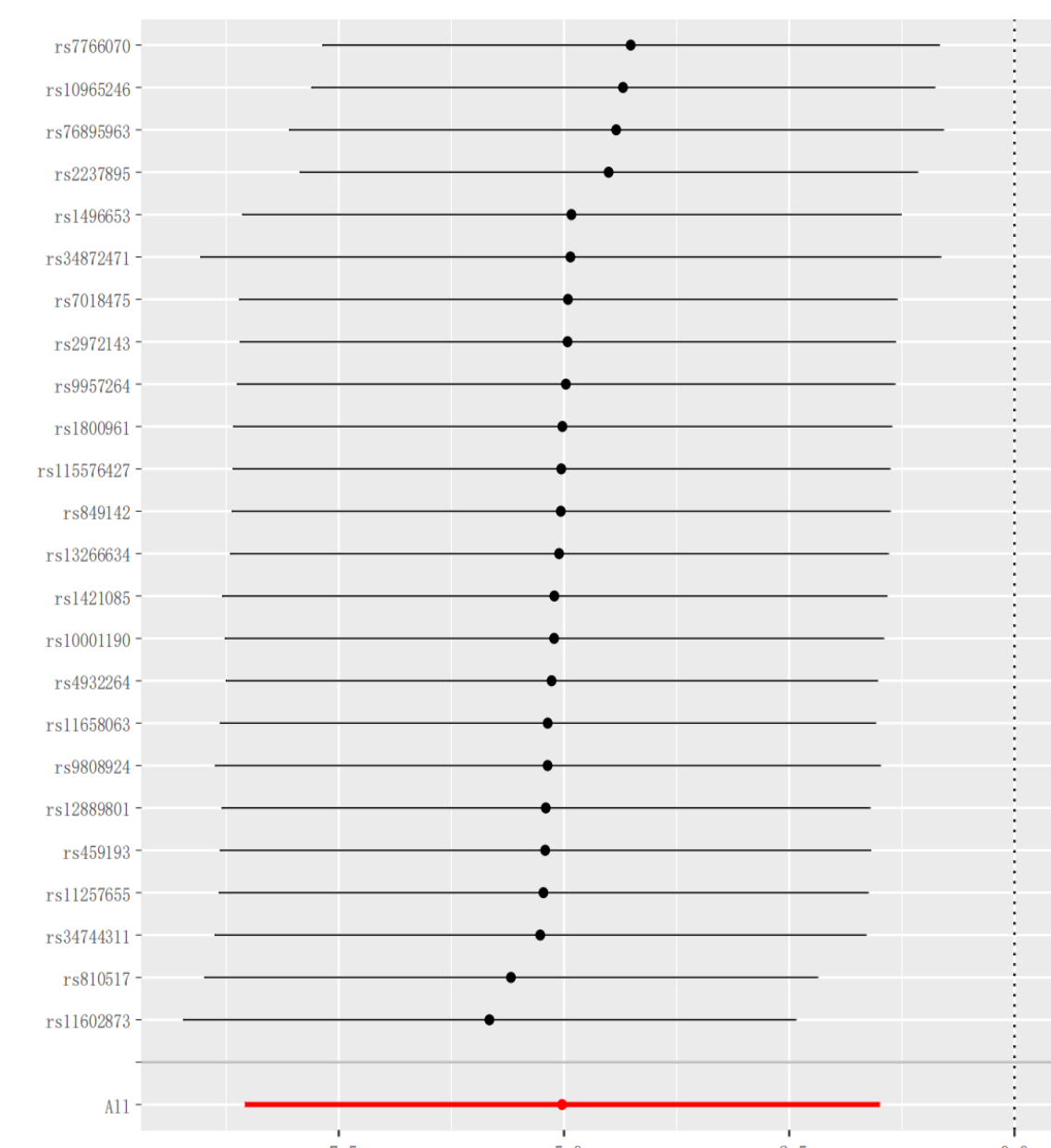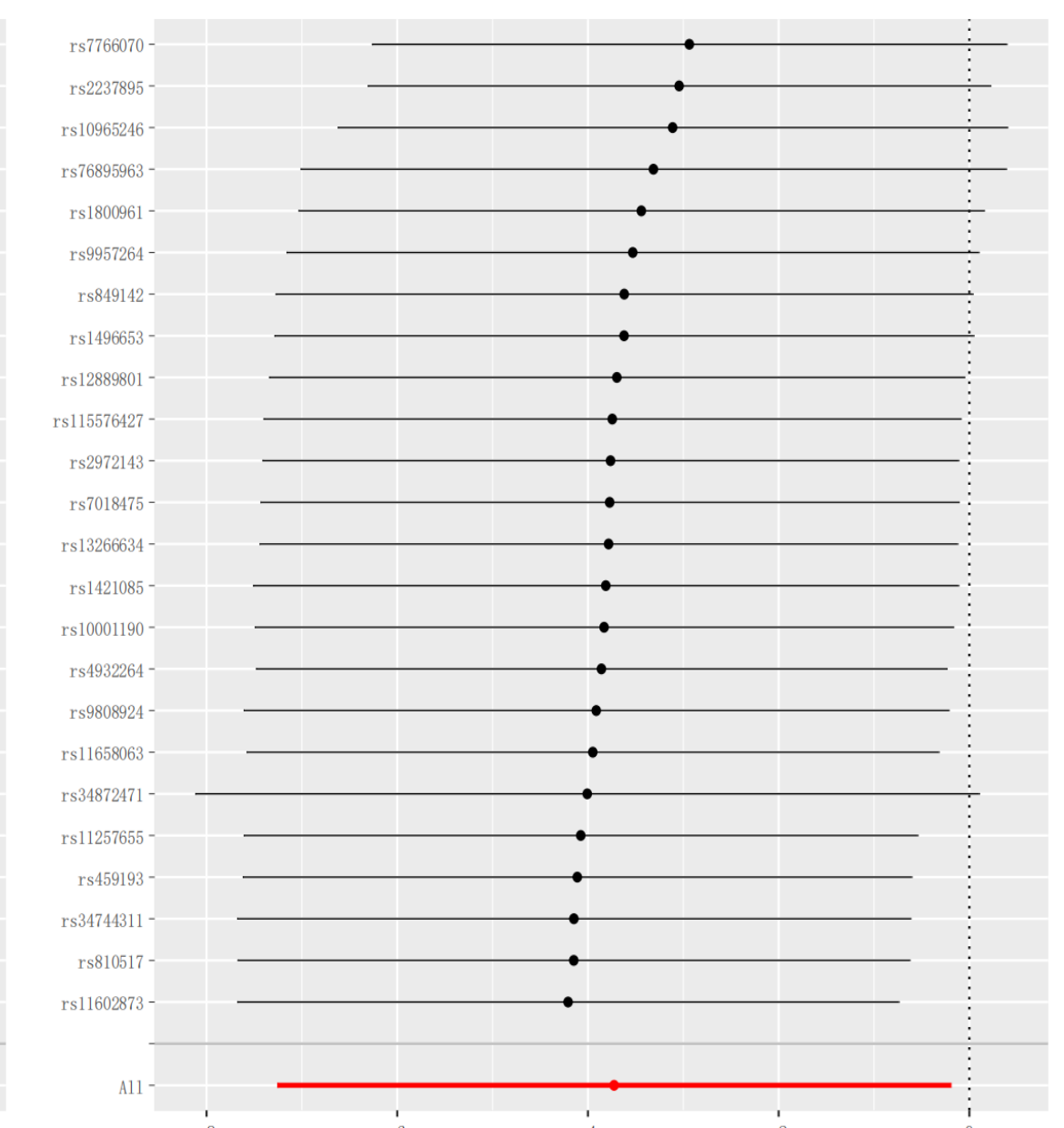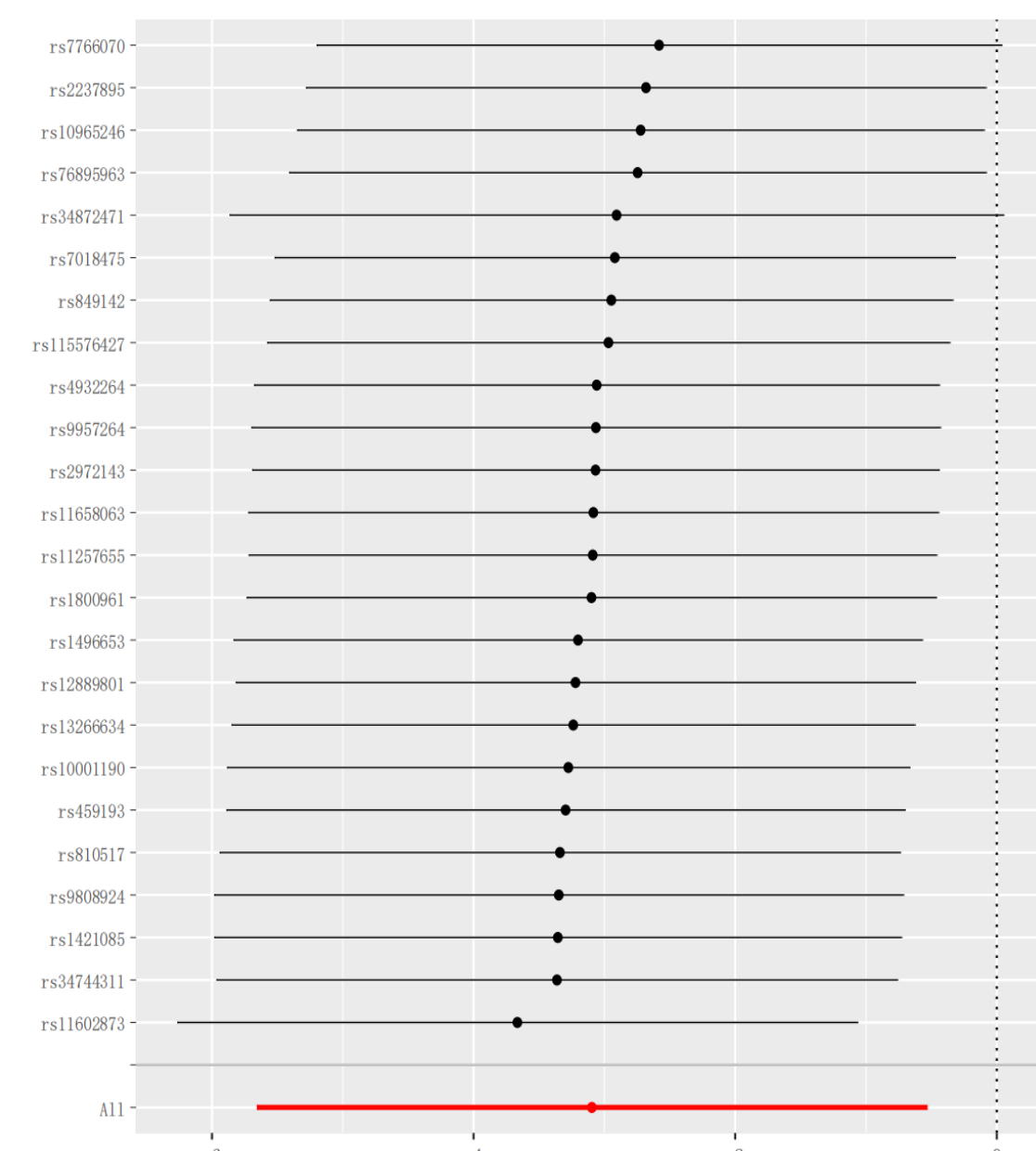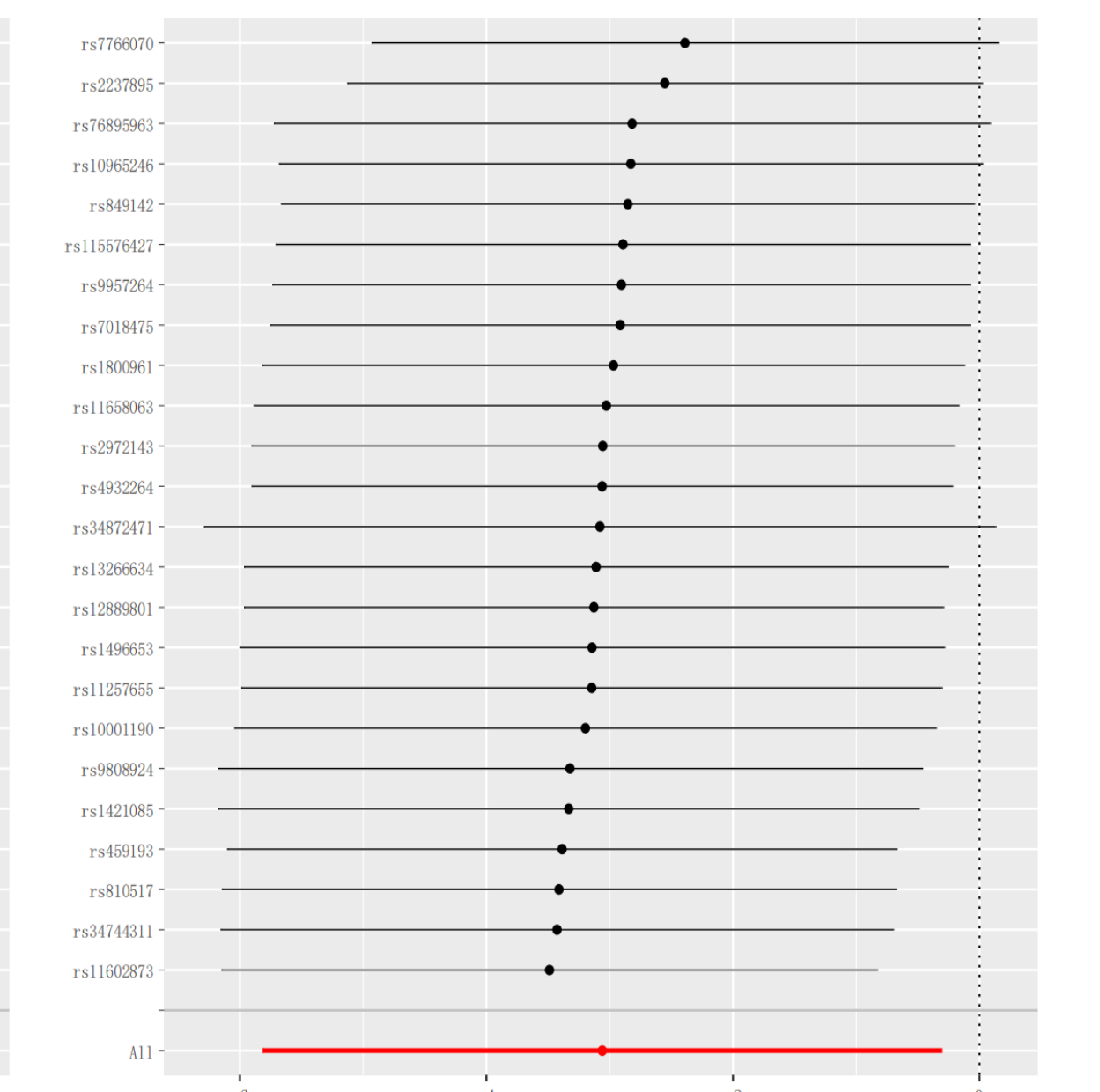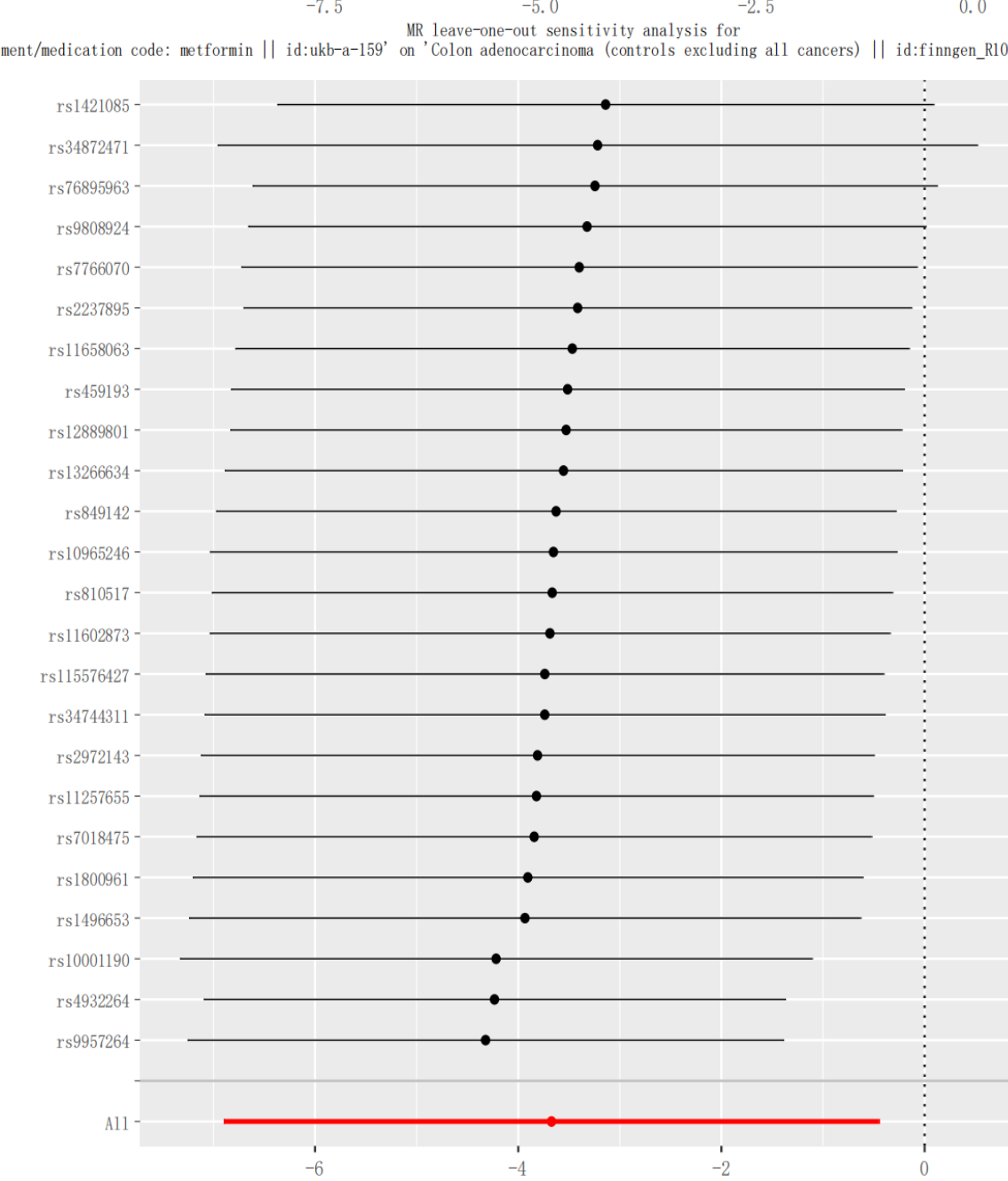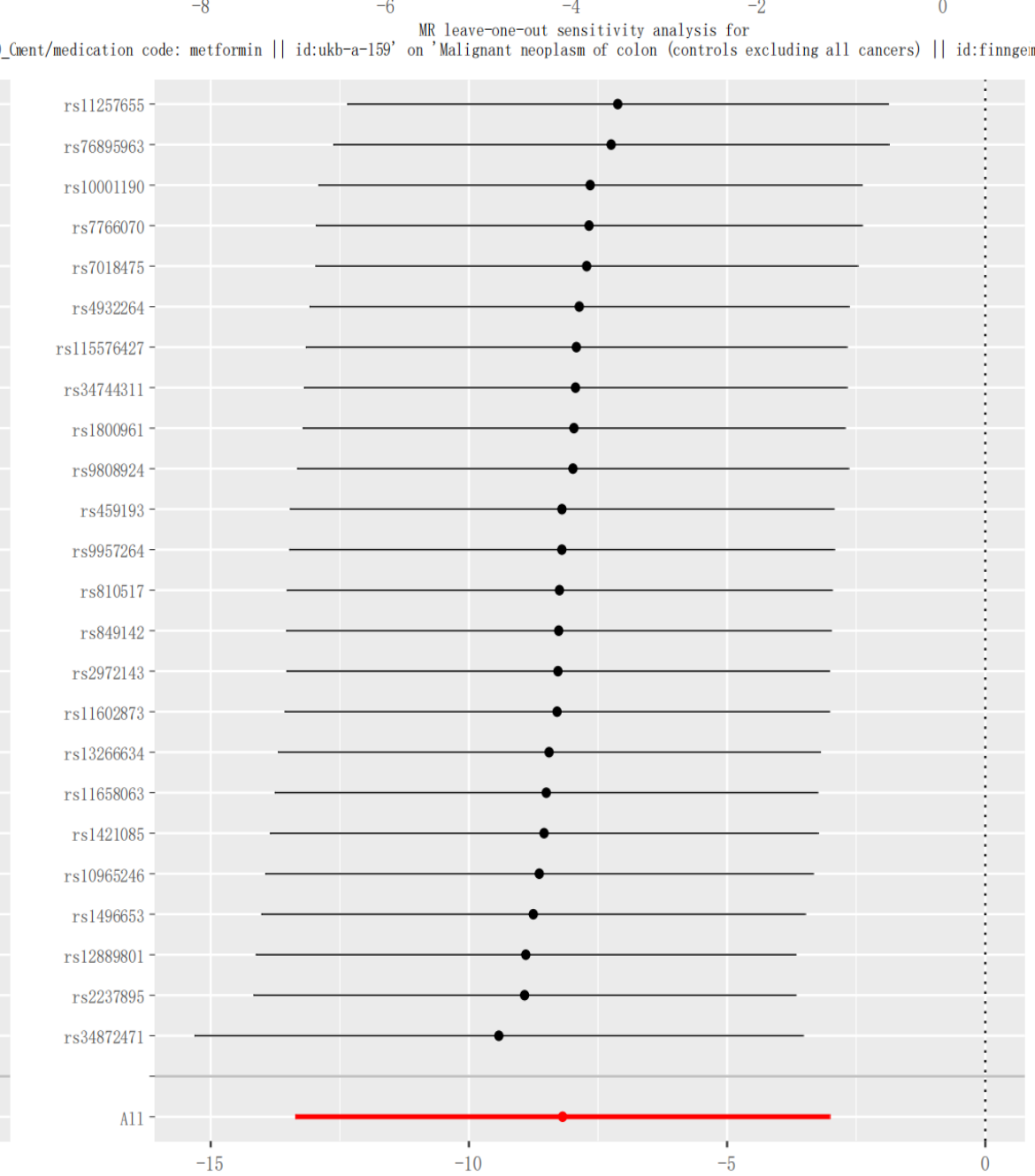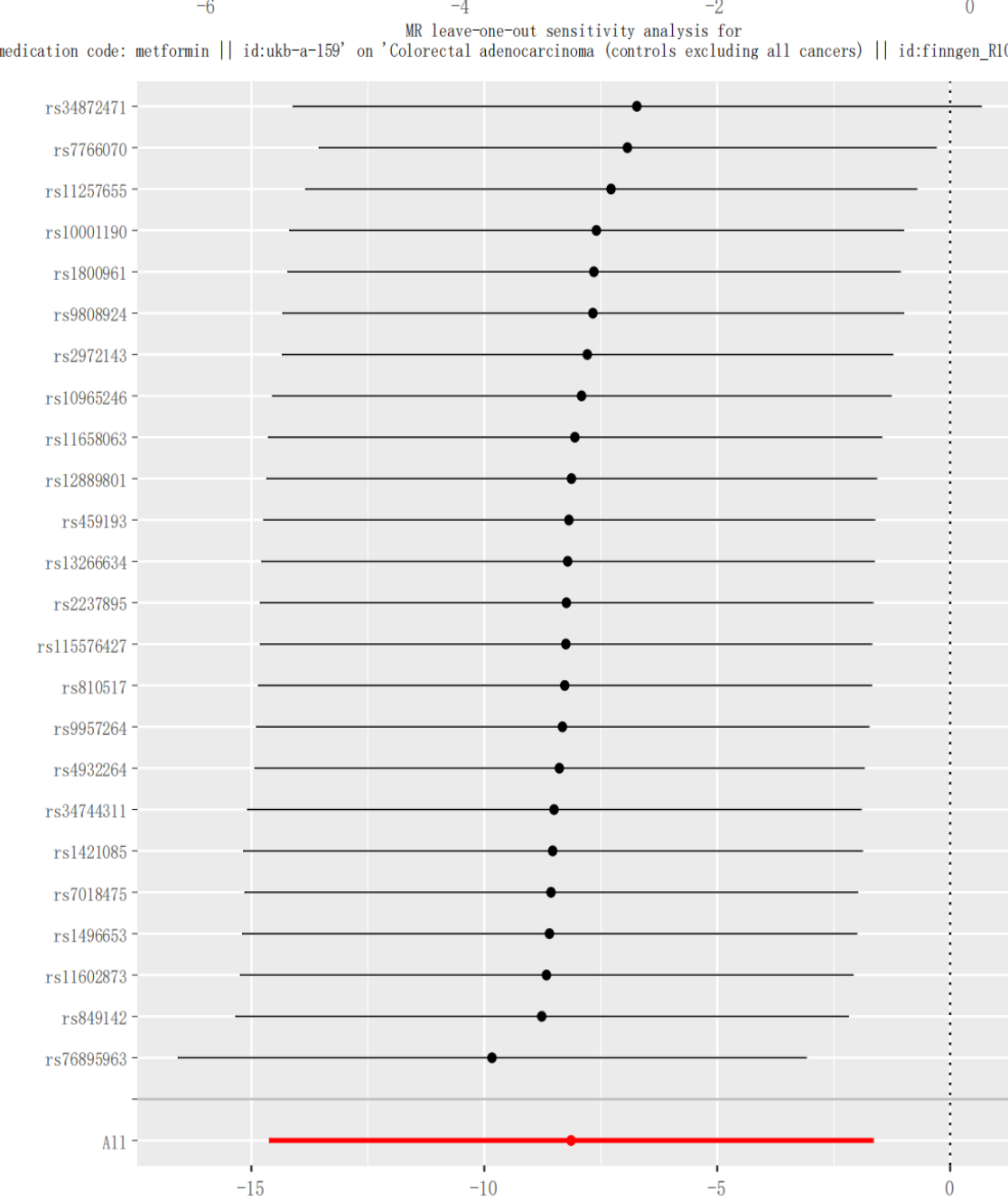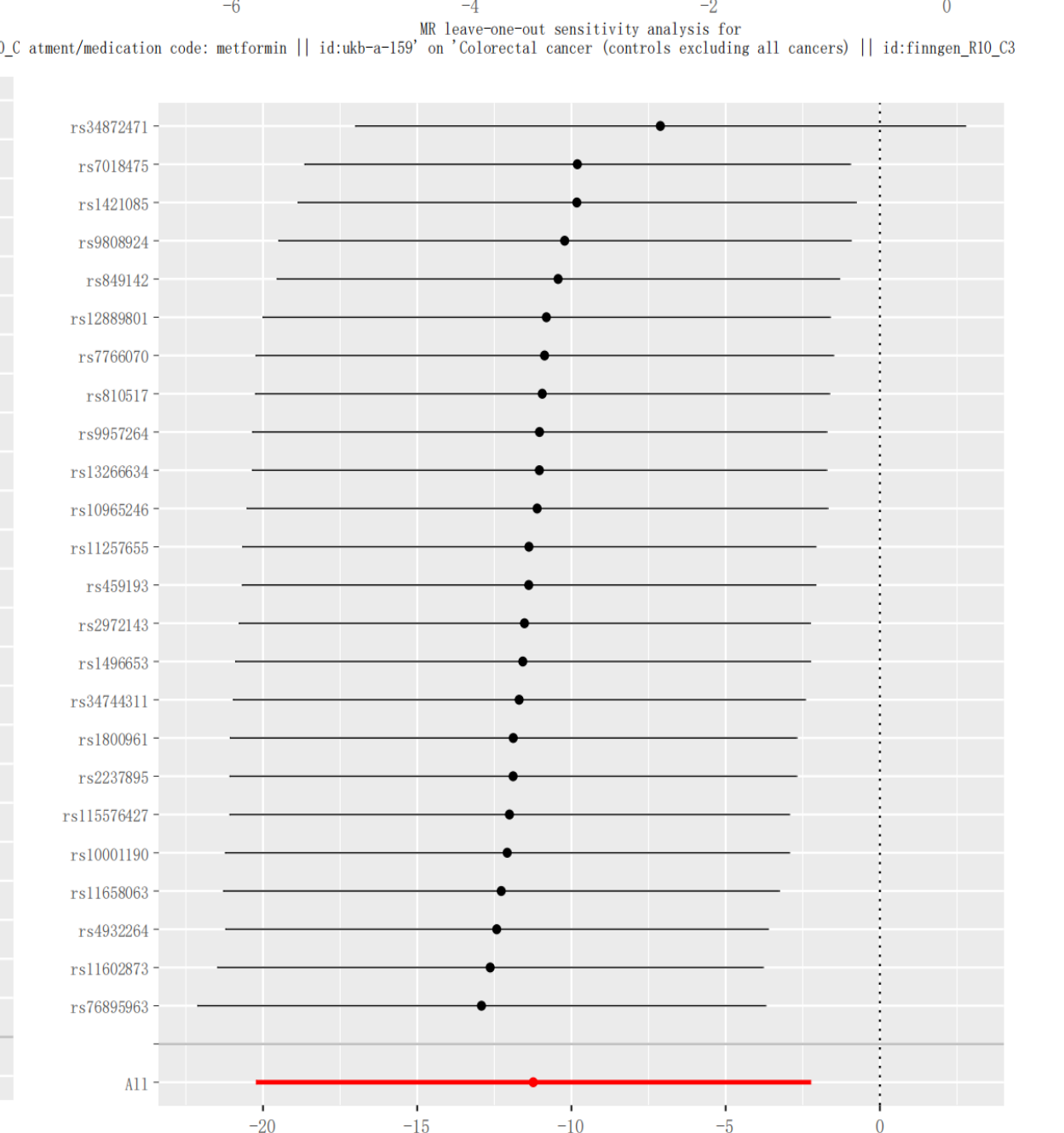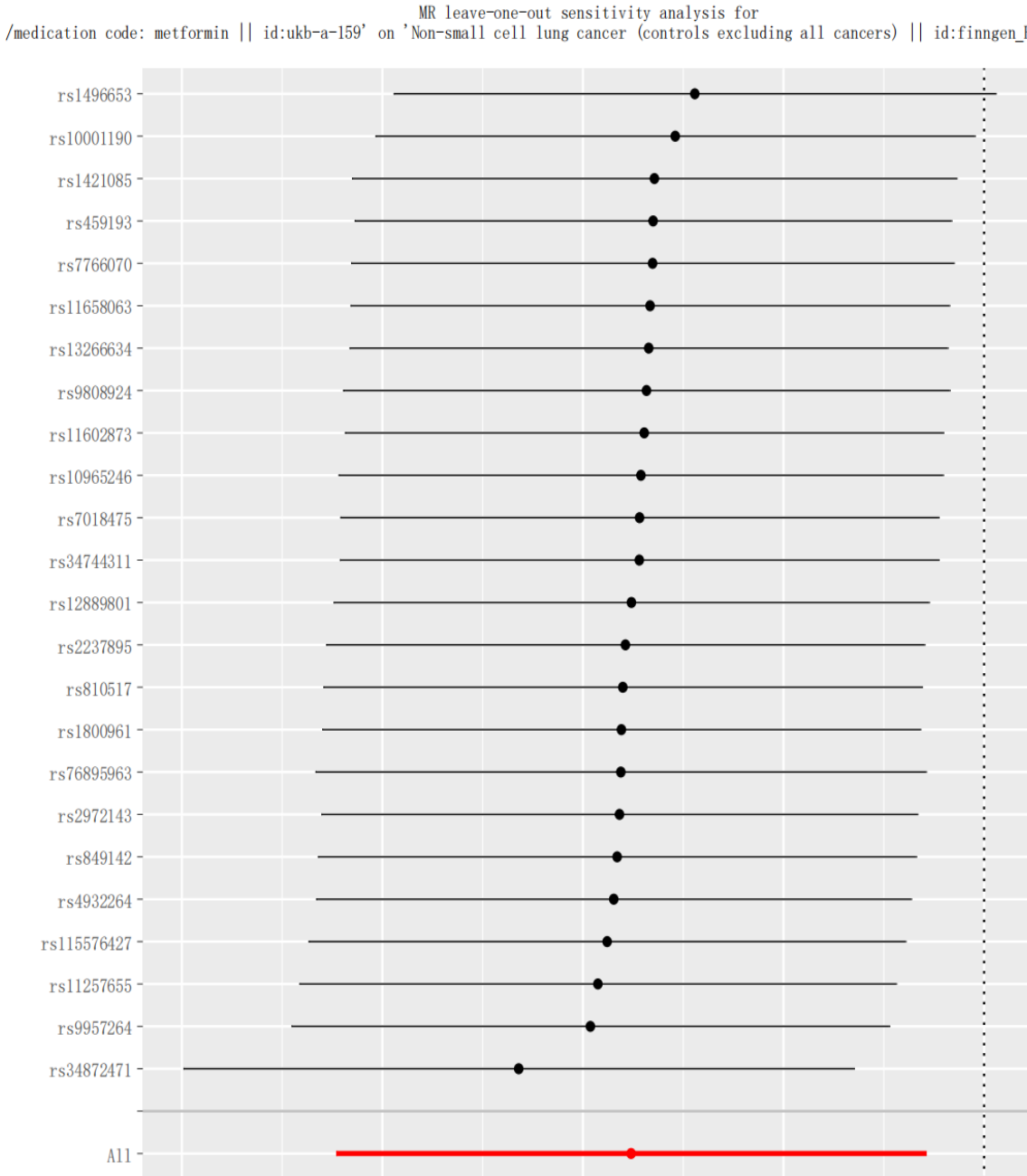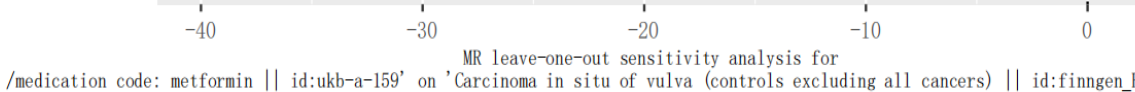

Supplement: Supplementary file 6 [file DataSheet8.PDF]
