# Supplementary material for: Bridging the gap: exploring the causal relationship between metformin and tumors
Source: Front Genet. 2024 Jun 19;15:1397390. doi: 10.3389/fgene.2024.1397390 (PMC11220117; doi:10.3389/fgene.2024.1397390)
Supplement: Supplementary file 5 [file DataSheet9.PDF]

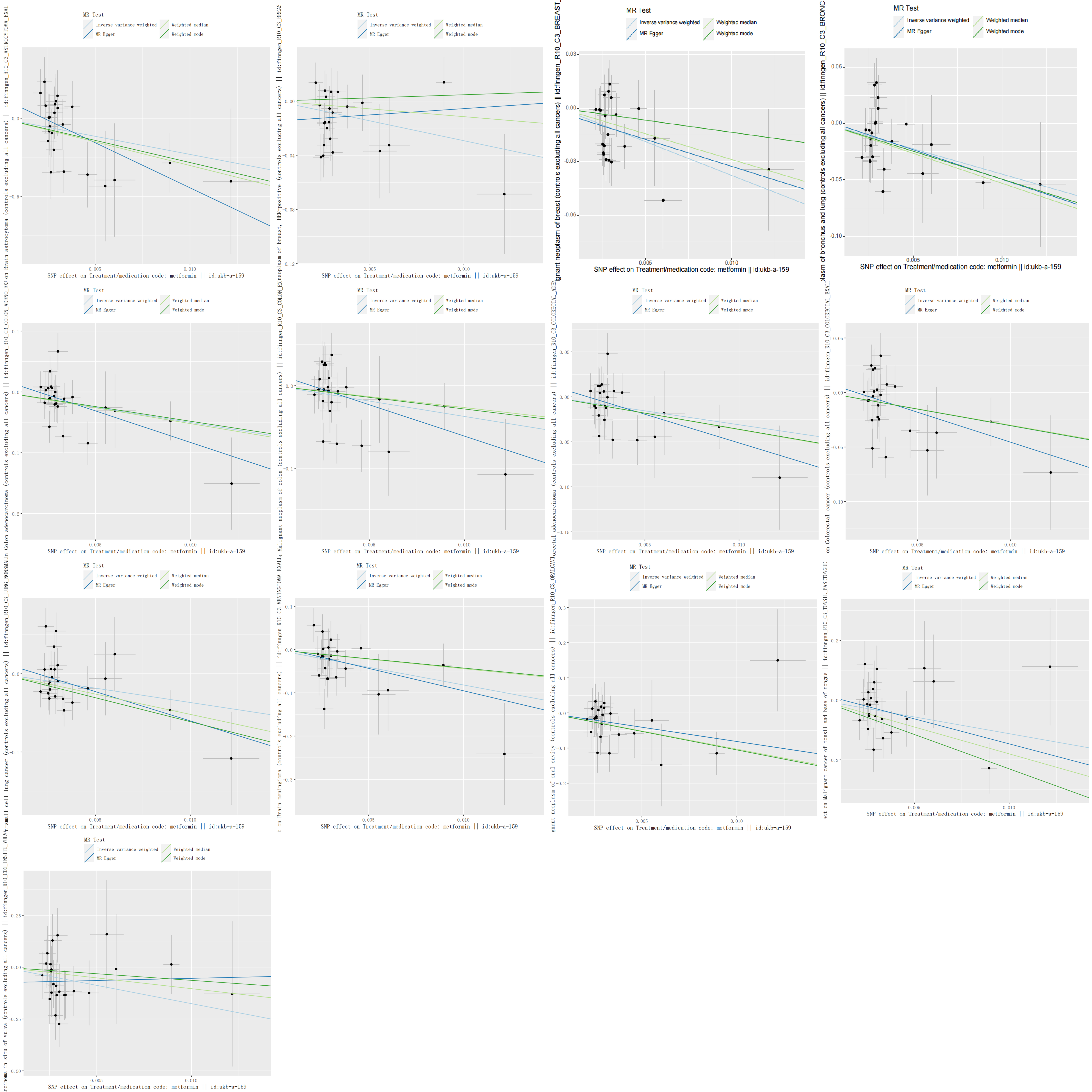
MR Test
Inverse variance weighted
Weighted median
MR Egger
Weighted mode
SNP effect on Treatment/medication code: metformin || id:ukb-a-159
on Brain astrocytoma (controls excluding all cancers) || id:finngen_R10_C3_ASTROCYTOMA_EXAL
neoplasm of breast, HER-positive (controls excluding all cancers) || id:finngen_R10_C3_BREAS
gnant neoplasm of breast (controls excluding all cancers) || id:finngen_R10_C3_BREAST_
lasm of bronchus and lung (controls excluding all cancers) || id:finngen_R10_C3_BRONC
Colon adenocarcinoma (controls excluding all cancers) || id:finngen_R10_C3_COLON_ADENO_EX
Malignant neoplasm of colon (controls excluding all cancers) || id:finngen_R10_C3_COLON_EX
orectal adenocarcinoma (controls excluding all cancers) || id:finngen_R10_C3_COLORECTAL_ADE
on Colorectal cancer (controls excluding all cancers) || id:finngen_R10_C3_COLORECTAL_EXAL
n-small cell lung cancer (controls excluding all cancers) || id:finngen_R10_C3_LUNG_NONSMAL
t on Brain meningioma (controls excluding all cancers) || id:finngen_R10_C3_MENINGIOMA_EXALL
gnant neoplasm of oral cavity (controls excluding all cancers) || id:finngen_R10_C3_ORALCAVI
ct on Malignant cancer of tonsil and base of tongue || id:finngen_R10_C3_TONSIL_BASETONGUE
rcinoma in situ of vulva (controls excluding all cancers) || id:finngen_R10_CD2_INSITU_VULV
